# Synthesis of tetrahydrofuro[3,2-*c*]pyridines via Pictet–Spengler reaction

**DOI:** 10.3762/bjoc.19.74

**Published:** 2023-06-30

**Authors:** Elena Y Mendogralo, Maxim G Uchuskin

**Affiliations:** 1 Perm State University, Bukireva st. 15, Perm, 614990, Russian Federation

**Keywords:** acid hydrolysis, 1,4-diketone, tetrahydrofuro[3,2-*c*]pyridines, Paal–Knorr reaction, Pictet–Spengler reaction

## Abstract

A semi-one-pot method for the synthesis of 4-substituted tetrahydrofuro[3,2-*c*]pyridines by the Pictet–Spengler reaction was developed. The method is based on the condensation of easily accessibly 2-(5-methylfuran-2-yl)ethanamine with commercially available aromatic aldehydes followed by acid-catalyzed Pictet–Spengler cyclization. Using this approach, we synthesized a range of 4-substituted tetrahydrofuro[3,2-*c*]pyridines in reasonable yields. The reactivity of some of the products was investigated and selected synthetic transformations of the obtained tetrahydrofuro[3,2-*c*]pyridines were shown.

## Introduction

**Figure 1:**
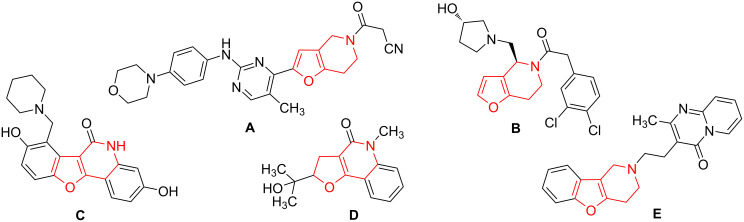
Examples of natural and bioactive hydrogenated furo[3,2-*c*]pyridines.

Hydrogenated furo[3,2-*c*]pyridines represent an important class of heterocyclic compounds, which skeleton is the key frame of many bioactive and natural compounds. For example, tetrahydrofuro[3,2-*c*]pyridine **A** demonstrates excellent in vitro JAK2 inhibitory activity superior to tofacitinib (Figure 1) [1]. Furan **B** is a potent κ-opioid receptor agonist and exhibits excellent antinociceptive activity [2]. Lactam **C** possesses potent antituberculosis activity and excellent selectivity to *Mycobacterium tuberculosis* strain H_37_Rv [3]. Araliopsine (**D**) was isolated from the fruits of *Zanthoxylum simulans* [4]. Benzofuran **E** is a potent and relatively selective α_2_-adrenoceptor antagonist [5].

**Scheme 1:**
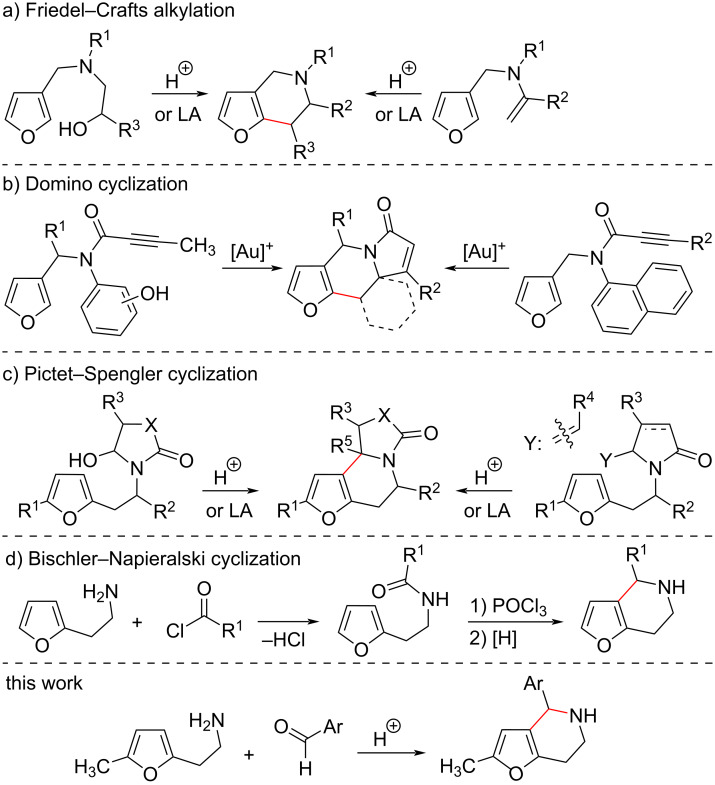
The described approaches to tetrahydrofuro[3,2-*c*]pyridines and our work.

Despite the simplicity of the tetrahydrofuro[3,2-*c*]pyridine core, only limited approaches for the synthesis of this subclass of heterocycles using furan derivatives as starting compounds have been described [6–8]. The first group of methods is based on the use of 3-substituted furans. For example, the intramolecular Friedel–Crafts alkylation reaction (Scheme 1a) of alcohols [9–11], alkenes [12] or acetylenes [13] affords the desired tetrahydrofuro[3,2-*c*]pyridines. A related method is based on a Au(I)-catalyzed domino sequence dearomatization/*ipso*-cyclization/Michael-type Friedel–Crafts alkylation (Scheme 1b) [14–16]. Unfortunately, approaches including the intramolecular alkylation of 3-substituted furans are underinvestigated as these substrates are usually hard to reach and the resulting benzyl carbocation is often prone to undergo rearrangement to a more stable benzhydryl-type cation resulting in the formation of isomeric products.

In an alternative group of methods, more accessible 2-substituted furans are used as starting compounds. For example, a construction of tetrahydrofuro[3,2-*c*]pyridines based on the Pictet–Spengler reaction [17–18] was described (Scheme 1c). The most studied variation of this cyclization is based on the generation of an acyliminium cation from the corresponding alcohols [19–23] or alkenes [24–29], subsequent attack of furan ring and the formation of annulated tetrahydrofuro[3,2-*c*]pyridines. Moreover, multistep cascade processes with the simultaneous construction of several cores were described, where the key step is the generation of an acyliminium cation and the Pictet–Spengler cyclization [30–32], including solid-phase synthesis [33–35]. Another route for the construction of tetrahydrofuro[3,2-*c*]pyridines is based on a Bischler–Napieralski cyclocondensation followed by the C=N bond reduction (Scheme 1d) [36–39]. Despite the simplicity of the latter approach and availability of 2-(furan-2-yl)ethanamine, the instability of intermediate dihydrofuro[3,2-*c*]pyridines, substantial tarring of reaction mixtures in acidic conditions and a limited number of cyclization examples might constrain its wide application.

Diverse bioactivities and prevalence of hydrogenated furo[3,2-*c*]pyridines in nature prompted us to develop an approach to substituted tetrahydrofuro[3,2-*c*]pyridines. From the analysis of the literature data [2,40–43], we suggested that the interaction of furanic amines with various aldehydes in an acidic media should be accompanied by the formation of the corresponding imine, the generation of the iminium cation, and subsequent Pictet–Spengler cyclization to form the key tetrahydrofuro[3,2-*c*]pyridines. For this method to be effective, the following requirements must be met: 1) the starting compounds must be commercially or readily available; 2) the resulting iminium cation should be stable enough for a smooth cyclization; 3) side processes and tarring of the reaction mixture should be minimized; 4) the possibility for the synthesis of a wide range of desired products should be realized. We have found that all requirements are met for the reaction of 2-(5-methylfuran-2-yl)ethanamine with a set of aromatic aldehydes. Herein, we report the results of our study of this tandem reaction.

## Results and Discussion

Initially, we synthesized the key imine **3a** by refluxing a solution of the starting 2-(5-methylfuran-2-yl)ethylamine (**1a**) and commercially available benzaldehyde (**2a**) in absolute CH_3_CN for one hour in a quantitative yield. To our delight, when 2 equiv of HCl were subsequently added to the solution of imine **3a** at 50 °C, the formation of the desired 2-methyl-4-phenyl-4,5,6,7-tetrahydrofuro[3,2-*c*]pyridine (**4a**) was observed in 26% yield (Table 1, entry 1). It should be noted that most part of the product **4a** was formed as the hydrochloride salt that was converted to the free base by the treatment with an aqueous solution of NaOH overnight (see Supporting Information File 1 for full experimental data). The yield of the desired product **4a** raised significantly when the reaction time was increased (Table 1, entry 2). At the same time, increasing the reaction temperature leads to the formation of the desired product **4a** with moderate yield (Table 1, entries 3 and 4). Next, we found that TsOH as a catalyst and several studied solvents (toluene, 1,4-dioxane, AcOH) are inefficient (Table 1, entries 5–10). Finally, we settled on the mixture of acetic and hydrochloric acid previously well-proven for furan chemistry [44–48]. Under these conditions at room temperature for 1 hour, the product yield was 18% (Table 1, entry 11). An increase of the reaction time up to 48 h leads to a significant increase of the yield (Table 1, entries 12 and 13). To reduce the reaction time, we increased the temperature (70 °C) and achieved a comparable result in 3.5 hours (Table 1, entry 14). Moreover, we found that the short-term treatment with aq NaOH solution at rt and at 50 °C overnight were less effective than the treatment with aq NaOH at rt overnight (Table 1, entries 15 and 16). Finally, we found that the optimal reaction time was 5 hours. The yield of the desired product **4a** in this case was 67% (Table 1, entry 18). With a shorter time, a slightly reduced reaction yield of **4a** was observed, while at longer heating the reaction mixture underwent abundant tarring (Table 1, entries 17 and 19). The treatment of the reaction mixture after the reaction with an aqueous solution of NaHCO_3_ turned out to be less effective (Table 1, entry 20). It should be noted that 3-(2-oxopropyl)-2-phenylpiperidin-4-one (**5a**), which is a product of further acid hydrolysis of **4a** was observed in most cases in trace amounts; an exception is entry 18 (Table 1), in which the 1,4-diketone **5a** was isolated in 10% yield.

**Table 1:** Optimization of reaction conditions^a^.

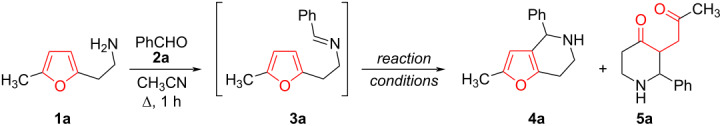					

Entry	Solvent	Time, h	*T*, °С	Acid, equiv	Yield of **4a**, %^b^

1	CH_3_CN	1.5	50	HCl (2.0)	26
2	CH_3_CN	5.5	50	HCl (1.0)	53
3	CH_3_CN	4.5	82	HCl (1.5)	32
4	CH_3_CN	24	82	HCl (1.5)	29
5	CH_3_CN	30	70	TsOH (2.0)	11
6	toluene	2	70	HCl (1.0)	58
7	toluene	2	110	HCl (1.0)	25
8	1,4-dioxane	2	70	HCl (1.0)	8
9	AcOH	24	50	TsOH (3.0)	13
10	AcOH	48	118	TsOH (4.0)	ND
11	AcOH	2	rt	HCl (1.0)	18
12	AcOH	24	rt	HCl (2.0)	31
13	AcOH	48	rt	HCl (2.0)	47
14	AcOH	3.5	70	HCl (2.0)	48
15^c^	AcOH	3.5	70	HCl (2.0)	41
16^d^	AcOH	3.5	70	HCl (2.0)	33
17	AcOH	1	70	HCl (2.0)	52
**18** ^e^	**AcOH**	**5**	**70**	**HCl (2.0)**	**67**
19	AcOH	6.5	70	HCl (2.5)	38
20^f^	AcOH	5	70	HCl (2.0)	50

^a^All reactions were performed at a 1 mmol scale of **1a**. The reaction mixture was treated with aq NaOH at rt overnight; ^b^isolated yield; ^c^treatment of the reaction mixture with aq NaOH at rt for 2 h; ^d^treatment of the reaction mixture with aq NaOH at 50 °C overnight; ^e^3-(2-oxopropyl)piperidin-4-one (**5a**) was isolated with 10% yield; ^f^treatment of the reaction mixture with aq NaHCO_3_ at rt overnight.

**Scheme 2:**
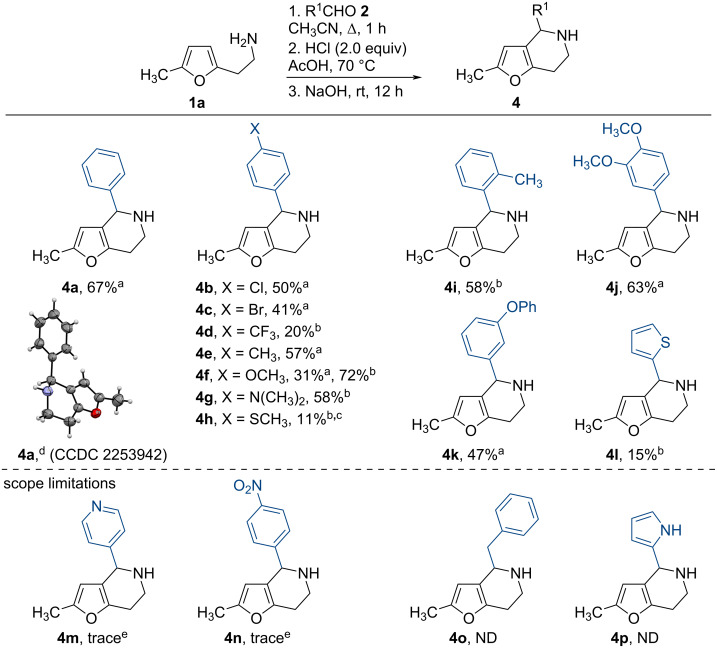
The synthesis of tetrahydrofuro[3,2-*c*]pyridines **4**. Conditions: ^a^the reaction was performed at 1 mmol scale of **1a**; ^b^the reaction was performed at 2 mmol scale of **1a**; ^c^the corresponding 3-(2-oxopropyl)piperidin-4-one was isolated with 20% yield; ^d^ORTEP diagram with ellipsoid contour probability level of 50%; ^e^detected by GC–MS.

With the optimized reaction conditions in hand, we investigated the scope of the developed tetrahydrofuro[3,2-*c*]pyridine synthesis. We found that a wide range of substituents at the aromatic cores was tolerant to the reaction conditions, and the yields of the desired products **4** remain moderate to good (Scheme 2). In particular, the use of starting benzaldehydes **2** containing electron-donating groups leads to the desired products **4e**–**g**,**i**–**k** in much higher yields than with substrate **4d** containing a strong electron-withdrawing group. The product **4n** with the nitro group in the *para*-position of starting benzaldehyde **2n** was formed in only trace amounts. A similar result was also observed in the case of isonicotinaldehyde (**2m**). These results were associated with an insufficient stabilization of the generated iminium cation due to the presence of a strong electron-withdrawing nitro group (for **3n**), and due to the fact that the electron deficiency of the pyridine ring is strongly manifested in an acidic media (for **3m**). In addition, the disadvantages of the developed method include the impossibility of using aliphatic aldehydes (for example, 2-phenylacetaldehyde (**2o**)), that is also explained by an insufficient stabilization of the resulting cation and the impossibility of cyclization. We also did not detect the formation of the target product when 1*H*-pyrrole-2-carbaldehyde (**2p**) was used. In this case, we observed abundant tarring and decomposition of the reaction mixture, which is probably due to the acidophobic nature of monosubstituted pyrroles. Surprisingly, the reaction of 4-(methylthio)benzaldehyde (**2h**) with 2-(5-methylfuran-2-yl)ethylamine (**1a**) under the optimized reaction conditions, in addition to the desired tetrahydrofuro[3,2-*c*]pyridine (**4h**), led to the major formation of the corresponding 1,4-diketone **5h**.

**Scheme 3:**
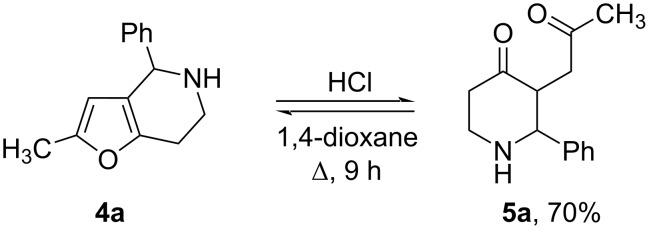
The acid-catalyzed reversible transformation of tetrahydrofuro[3,2-*c*]pyridine **4a** and 3-(2-oxopropyl)piperidin-4-one **5a**. The product yield was determined by GC–MS using an internal standard.

This unexpected formation of 3-(2-oxopropyl)piperidin-4-one (**5h**) led us to the idea that the tandem sequence Pictet–Spengler cyclization/furan acid hydrolysis could be a convenient tool for the synthesis of substituted 3-(2-oxopropyl)piperidin-4-ones **5**. Using tetrahydrofuro[3,2-*c*]pyridine **4a** as the model compound, we studied the effect of various Brønsted acids, temperature, concentrations, and the nature of the solvent on the efficiency of the reaction. In most cases, we observed the abundant tarring of the reaction mixture and we could not achieve the full conversion of the starting tetrahydrofuro[3,2-*c*]pyridine **4a** in any experiment. The best results were achieved when HCl in 1,4-dioxane was used (Scheme 3). However, upon isolation and purification of the desired product by column chromatography, as well as upon recrystallization from ethyl acetate, 1,4-diketone **5а** undergoes Paal–Knorr cyclization with the formation of the starting tetrahydrofuro[3,2-*c*]pyridine **4а**, which ultimately leads to mixtures of compounds **4а** and **5а** with different ratios. The reversibility of the acid hydrolysis is quite typical for furans and is more pronounced for di- and polysubstituted furans, due to the higher stability of the latter [49–51].

**Scheme 4:**
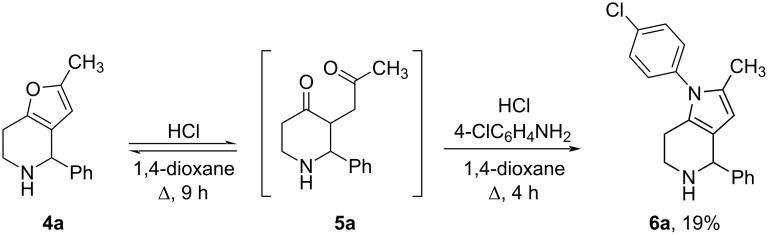
Synthesis of tetrahydropyrrolo[3,2-*c*]pyridine **6a**.

3-(2-Oxopropyl)piperidin-4-one **5a** could be involved into the Paal–Knorr pyrrole synthesis in a one-pot manner. Thus, without isolation of the intermediate 3-(2-oxopropyl)piperidin-4-one **5a**, the reaction mixture after acid hydrolysis was treated with aniline and the desired tetrahydro-1*H*-pyrrolo[3,2-*с*]pyridine **6а** was isolated in 19% yield only (Scheme 4).

**Scheme 5:**
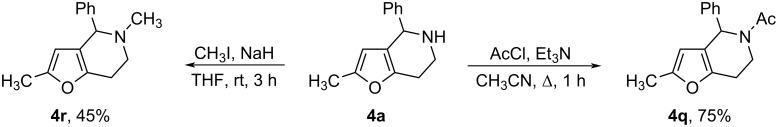
Reactivity of tetrahydrofuro[3,2-*c*]pyridine **4a**.

Finally, we studied some reactions of the obtained tetrahydrofuro[3,2-*c*]pyridine **4a**. We showed that the *N*-acetyl-protected tetrahydrofuro[3,2-*c*]pyridine **4q** could be obtained by treating the starting tetrahydrofuro[3,2-*c*]pyridine **4a** with acetyl chloride while the *N*-methylated tetrahydrofuro[3,2-*c*]pyridine **4r** can be formed via subsequent treatment of **4a** with NaH and methyl iodide (Scheme 5).

## Conclusion

In conclusion, we have developed a semi-one-pot method for the synthesis of 2,3-annulated furans based on the Pictet–Spengler reaction. This method provides a shortcut to 4-substituted tetrahydrofuro[3,2-*c*]pyridines in reasonable yields starting from readily available 2-(5-methylfuran-2-yl)ethylamine and aromatic aldehydes. The use of starting benzaldehydes containing electron-donating groups leads to the desired products in much higher yields than with aldehydes containing electron-withdrawing moieties. The availability of the starting materials, mild reaction conditions, and reasonable yields are some of the attractive features of the developed method. In addition, we have also demonstrated some transformations of the products into functionalized derivatives.

## Supporting Information

File 1 Experimental procedures, characterization data, copies of ^1^H and ^13^C NMR spectra, HRMS of new compounds, and X-ray crystallography data.

File 2 Crystallographic information file for compound **4a**.
