# Supplementary material for: Synthesis of tetrahydrofuro[3,2-c]pyridines via Pictet–Spengler reaction
Source: Beilstein J Org Chem. 2023 Jun 30;19:991–7. doi: 10.3762/bjoc.19.74 (PMC10315887; doi:10.3762/bjoc.19.74)
Supplement: File 1 — Experimental procedures, characterization data, copies of 1H and 13C NMR spectra, HRMS of new compounds, and X-ray crystallography data. [file Beilstein_J_Org_Chem-19-991-s001.pdf]

BEILSTEIN JOURNAL OF ORGANIC CHEMISTRY

Supporting Information

for

Synthesis of tetrahydrofuro[3,2-*c*]pyridines via Pictet–Spengler reaction

Elena Y. Mendogralo and Maxim G. Uchuskin

Beilstein J. Org. Chem. **2023,** *19,* 991–997. doi:10.3762/bjoc.19.74

Experimental procedures, characterization data, copies of ^{1}H and ^{13}C NMR spectra, HRMS of new compounds, and X-ray crystallography data

License and Terms: This is a supporting information file under the terms of the Creative Commons Attribution License (https://creativecommons.org/licenses/by/4.0). Please note that the reuse, redistribution and reproduction in particular requires that the author(s) and source are credited and that individual graphics may be subject to special legal provisions.
The license is subject to the *Beilstein Journal of Organic Chemistry* terms and conditions: (https://www.beilstein-journals.org/bjoc/terms)

Table of contents

1. General information S2
2. General procedure for the synthesis of tetrahydrofuro[3,2-*c*]pyridines **4** S2
3. Synthesis of 3-(2-oxopropyl)-2-phenylpiperidin-4-one (**5a**) S8
4. Synthesis of 1-(4-chlorophenyl)-2-methyl-4-phenyl-4,5,6,7-tetrahydro-1*H*-pyrrolo[3,2-*c*]pyridine (**6a**) S8
5. Synthesis of 2,5-dimethyl-4-phenyl-4,5,6,7-tetrahydrofuro[3,2-*c*]pyridine (**4r**) S9
6. Synthesis of 1-(2-methyl-4-phenyl-6,7-dihydrofuro[3,2-*c*]pyridin-5(4*H*)-yl)ethan-1-one (**4q**) S10
7. Copies of ^{1}H, ^{13}C NMR spectra of new compounds S11
8. X-ray crystallography data S28
9. References S30

1. General information

^{1}H and ^{13}C NMR spectra were recorded on a Bruker Avance III HD 400 (400 MHz for ^{1}H and 100 MHz for ^{13}C NMR) at 40 °C. The chemical shifts (δ) were measured in ppm with respect to the solvent ([D_6] DMSO, ^{1}H: δ = 2.50 ppm, ^{13}C: δ = 39.52 ppm). Coupling constants (*J*) are given in hertz (Hz). The peak patterns are indicated as follows: s (singlet), d (doublet), t (triplet), q (quartet), m (multiplet), dd (doublet of doublets) and br (broadened). High-resolution mass measurements (HRMS) were carried out using a Bruker microTOF-QTM ESI-TOF mass spectrometer. GC/MS analysis was performed on an Agilent 7890B interfaced to an Agilent 5977A mass selective detector. Melting points were determined with a Stuart SMP 30. Data sets for X-ray diffraction were collected with a New Xcalibur, Ruby diffractometer. Column chromatography was performed on silica gel Macherey Nagel (40–63 μm). Flash column chromatography was performed over silica gel (0.04–0.063 mm) using a mixture of ethyl acetate and petroleum ether. TLC plates were visualized by exposure to ultraviolet light. Starting 2-(5-methylfuran-2-yl)ethanamine was synthesized according to known procedure [1]. All the reactions were carried out using freshly distilled and dry solvents from solvent stills.

2. General procedure for the synthesis of tetrahydrofuro[3,2-*c*]pyridines 4.

Method A: To a solution of aldehyde **2** (1.0 mmol) in dry acetonitrile (1 mL) 2-(5-methylfuran-2-yl)ethanamine (**1a**, 1.0 mmol, 125 μL) was added. The reaction mixture was heated at 82 °C for 1 h (TLC control) and concentrated to dryness. To the solution of crude imine in glacial AcOH (750 μL) was added portionwise conc. HCl (500 μL). The reaction mixture was stirred at 70 °C for 5 h (TLC control). Then an aq. saturated solution of NaOH was added and the mixture was stirred overnight at room temperature. The formed precipitate was filtered and the filtrate was extracted with

ethyl acetate (3 × 10 mL). The combined organic layers were washed with brine (3 × 10 mL), dried with anhydrous Na_2SO_4 and concentrated to dryness. The residue and precipitate were combined. The product was purified by column chromatography on silica gel using a mixture of petroleum ether/ethyl acetate 1:1 as an eluent and recrystallized from a suitable solvent.

Method B: To a solution of aldehyde **2** (2.0 mmol) in dry acetonitrile (2 mL) 2-(5-methylfuran-2-yl)ethanamine (**1a**, 2.0 mmol, 250 µL) was added. The reaction mixture was heated at 82 °C for 1 h (TLC control) and concentrated to dryness. To the solution of the crude imine in glacial AcOH (1.5 mL) was added portionwise conc. HCl (1 mL). The reaction mixture was stirred at 70 °C for 5 h (TLC control). Then aq. saturated solution of NaOH was added and the mixture was stirred overnight at room temperature. The formed precipitate was filtered and the filtrate was extracted with ethyl acetate (3 × 20 mL). The combined organic layers were washed with brine (3 × 20 mL), dried with anhydrous Na_2SO_4 and concentrated to dryness. The residue and precipitate were combined. The product was purified by column chromatography on silica gel using a mixture of petroleum ether/ethyl acetate 1:1 as an eluent and recrystallized from a suitable solvent.

*2-Methyl-4-phenyl-4,5,6,7-tetrahydrofuro[3,2-c]pyridine (**4a**)* [2]. Yield: 143 mg, 67% (method A); pale beige solid; mp = 107–108 °C (petroleum ether/acetone); ^{1}H NMR (400 MHz, DMSO-d_6): δ 7.31 – 7.22 (m, 5H), 5.60 (br. s, 1H), 4.75 (br. t, J = 1.9 Hz, 1H), 3.13 – 3.07 (m, 1H), 2.95 – 2.88 (m, 1H), 2.67 – 2.60 (m, 1H), 2.52 – 2.48 (m, 1H), 2.19 (s, 3H) ppm; ^{13}C{^{1}H} NMR (100 MHz, DMSO-d_6): δ 149.0, 147.5, 143.7, 127.9 (2C), 127.7 (2C), 126.7, 120.2, 104.9, 56.2, 41.5, 24.1, 13.1 ppm; HRMS (ESI$^+$) m/z: [M+H]$^+$ Calcd for $C_{14}H_{16}NO^+$ 214.1226; found 214.1225.

*3-(2-Oxopropyl)-2-phenylpiperidin-4-one (**5a**).* Product **5a** was obtained by method A, along with the major product **4a**. Yield: 23 mg, 10% (dr > 19:1 determined by NMR); pale beige oil; ^{1}H NMR (400 MHz, DMSO-d_6): δ 7.40 – 7.34 (m, 5H), 3.57 (s, 1H), 3.54 (s, 1H), 3.34 – 3.29 (m, 1H), 3.05 – 2.99 (m, 1H), 2.84 – 2.78 (m, 1H), 2.70 – 2.58 (m, 3H), 2.27 – 2.23 (m, 1H), 1.94 (s, 3H) ppm; ^{13}C{^{1}H} NMR (100 MHz, DMSO-d_6): δ 208.5, 206.3, 141.9, 129.2, 128.4 (2C), 127.6 (2C), 66.5, 53.9, 46.2, 42.5, 38.9, 30.0 ppm; HRMS (ESI$^+$) m/z: [M+H]$^+$ Calcd for $C_{14}H_{18}NO_2^+$ 232.1332; found 232.1330.

*2-Methyl-4-phenyl-4,5,6,7-tetrahydrofuro[3,2-c]pyridine hydrochloride (**4a’**).* Product **4a’** was obtained by method A, along with the major product **4a**. Yield: 20 mg, 8%; yellow oil; ^{1}H NMR (400 MHz, DMSO-d_6): δ 7.32 – 7.25 (m, 5H), 5.61 (br. s, 1H), 4.77 (br. s, 1H), 4.08 (br. s, 2H), 3.13 – 3.09 (m, 1H), 2.96 – 2.91 (m, 1H), 2.68 – 2.61 (m, 1H), 2.54 – 2.50 (m, 1H), 2.19 (s, 3H) ppm; ^{13}C{^{1}H} NMR (100 MHz, DMSO-d_6): δ 149.0, 147.5, 143.5, 127.9 (2C), 127.7 (2C), 126.8, 120.1, 104.9, 56.2, 41.5, 24.0, 13.1 ppm.

*4-(4-Chlorophenyl)-2-methyl-4,5,6,7-tetrahydrofuro[3,2-c]pyridine (**4b**).* Yield: 124 mg, 50% (method A); yellow solid; mp = 73–75 °C (petroleum ether/ethyl acetate); ^{1}H NMR (400 MHz, DMSO-d_6): δ 7.37 – 7.31 (m, 4H), 5.62 (br. s, 1H), 4.75 (br. s, 1H), 3.09 – 3.04 (m, 1H), 2.94 – 2.88 (m, 1H), 2.65 – 2.58 (m, 2H), 2.52 – 2.48 (m, 1H), 2.19 (s, 3H) ppm; ^{13}C{^{1}H} NMR (100 MHz, DMSO-d_6): δ 149.1, 147.6, 142.8, 131.3, 129.5 (2C), 127.8 (2C), 119.8, 104.7, 55.4, 41.3, 24.0, 13.1 ppm; HRMS (ESI$^+$) m/z: [M+H]$^+$ Calcd for $C_{14}H_{15}ClNO^+$ 248.0837; found 248.0846.

*4-(4-Bromophenyl)-2-methyl-4,5,6,7-tetrahydrofuro[3,2-c]pyridine (**4c**).* Yield: 120 mg, 41% (method A); yellow oil; ^{1}H NMR (400 MHz, DMSO-d_6): δ 7.49 (d, J = 8.3 Hz, 2H),

7.27 (d, J = 8.3 Hz, 2H), 5.62 (br. s, 1H), 4.74 (br. s, 1H), 3.09 – 3.04 (m, 1H), 2.94 – 2.88 (m, 1H), 2.65 – 2.58 (m, 1H), 2.51 – 2.48 (m, 1H), 2.18 (s, 3H) ppm; $^{13}C\{^{1}H\}$ NMR (100 MHz, DMSO-d_6): δ 149.2, 147.6, 143.2, 130.8 (2C), 130.0 (2C), 119.8, 119.7, 104.7, 55.4, 41.3, 24.0, 13.1 ppm; HRMS (ESI$^+$) m/z: [M+H]$^+$ Calcd for $C_{14}H_{15}BrNO^+$ 292.0332; found 292.0326.

*2-Methyl-4-[4-(trifluoromethyl)phenyl]-4,5,6,7-tetrahydrofuro[3,2-c]pyridine (**4d**).* Yield: 112 mg, 20% (method B); pale beige solid; mp = 87 °C (petroleum ether/ethyl acetate); ^{1}H NMR (400 MHz, DMSO-d_6): δ 7.67 (d, J = 8.2 Hz, 2H), 7.54 (d, J = 8.2 Hz, 2H), 5.65 (br. s, 1H), 4.85 (br. s, 1H), 3.10 – 3.05 (m, 1H), 2.97 – 2.91 (m, 1H), 2.75 (br. s, 1H), 2.67 – 2.60 (m, 1H), 2.55 – 2.53 (m, 1H), 2.19 (s, 3H) ppm; $^{13}C\{^{1}H\}$ NMR (100 MHz, DMSO-d_6): δ 149.3, 148.6 (br. s), 147.7, 128.5 (2C), 127.5 (q, $^{2}J_{CF}$ = 31 Hz), 124.8 (q, $^{3}J_{CF}$ = 3.8 Hz, 2C), 124.3 (q, $^{1}J_{CF}$ = 270 Hz), 119.5, 104.7, 55.6, 41.3, 24.0, 13.1 ppm; HRMS (ESI$^+$) m/z: [M+H]$^+$ Calcd for $C_{15}H_{15}F_3NO^+$ 282.1100; found 282.1095.

*2-Methyl-4-(4-methylphenyl)-4,5,6,7-tetrahydrofuro[3,2-c]pyridine (**4e**).* Yield: 129 mg, 57% (method A); yellow oil; ^{1}H NMR (400 MHz, DMSO-d_6): δ 7.17 (d, J = 8.0 Hz, 2H), 7.10 (d, J = 8.0 Hz, 2H), 5.57 (s, 1H), 4.69 (br. s, 1H), 3.11 – 3.06 (m, 1H), 2.92 – 2.86 (m, 1H), 2.65 – 2.58 (m, 1H), 2.50 – 2.46 (m, 1H), 2.28 (s, 3H), 2.17 (s, 3H) ppm; $^{13}C\{^{1}H\}$ NMR (100 MHz, DMSO-d_6): δ 148.9, 147.5, 140.7, 135.8, 128.4 (2C), 127.6 (2C), 120.4, 104.9, 56.0, 41.5, 24.1, 20.5, 13.1 ppm; HRMS (ESI$^+$) m/z: [M+H]$^+$ Calcd for $C_{15}H_{18}NO^+$ 228.1383; found 228.1386.

*4-(4-Methoxyphenyl)-2-methyl-4,5,6,7-tetrahydrofuro[3,2-c]pyridine (**4f**).* Yield: 350 mg, 72% (method B); yellow oil; ^{1}H NMR (400 MHz, DMSO-d_6): δ 7.19 (d, J = 8.6 Hz,

2H), 6.85 (d, J = 8.6 Hz, 2H), 5.57 (s, 1H), 4.68 (s, 1H), 3.73 (s, 3H), 3.10 – 3.05 (m, 1H), 2.91 – 2.85 (m, 1H), 2.64 – 2.57 (m, 1H), 2.50 – 2.46 (m, 1H), 2.18 (s, 3H) ppm; $^{13}C\{^1H\}$ NMR (100 MHz, DMSO-d_6): δ 158.2, 148.9, 147.5, 135.8, 128.8 (2C), 120.5, 113.3 (2C), 104.9, 55.6, 54.9, 41.5, 24.1, 13.1 ppm; HRMS (ESI$^+$) m/z: $[M+H]^+$ Calcd for $C_{15}H_{18}NO_2^+$ 244.1332; found 244.1328.

*N,N-Dimethyl-4-(2-methyl-4,5,6,7-tetrahydrofuro[3,2-c]pyridin-4-yl)aniline (**4g**).* Yield: 297 mg, 58% (method B); pale beige solid; mp = 74–76 °C (petroleum ether/ethyl acetate); ^1H NMR (400 MHz, DMSO-d_6): δ 7.08 (d, J = 8.8 Hz, 2H), 6.66 (d, J = 8.8 Hz, 2H), 5.56 (s, 1H), 4.62 (br. s, 1H), 3.11 – 3.05 (m, 1H), 2.90 – 2.87 (m, 1H), 2.86 (s, 6H), 2.64 – 2.57 (m, 1H), 2.46 – 2.44 (m, 1H), 2.18 (s, 3H) ppm; $^{13}C\{^1H\}$ NMR (100 MHz, DMSO-d_6): δ 150.2, 149.3, 148.0, 131.9, 128.9 (2C), 121.3, 112.6 (2C), 105.6, 56.3, 42.1, 40.8 (2C), 24.7, 13.7 ppm; HRMS (ESI$^+$) m/z: $[M+H]^+$ Calcd for $C_{16}H_{21}N_2O^+$ 257.1648; found 257.1651.

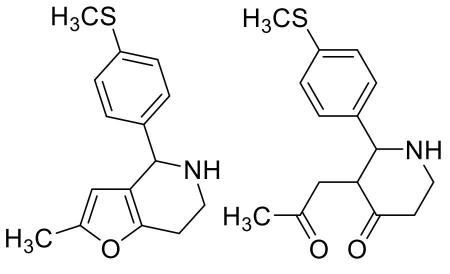

4h **5h**

*Mixture of 2-methyl-4-[4-(methylthio)phenyl]-4,5,6,7-tetrahydrofuro[3,2-c]pyridine (**4h**) and 2-[4-(methylthio)phenyl]-3-(2-oxopropyl)piperidin-4-one (**5h**).* **4h** yield: 55 mg, 11% (method B); **5h** yield: 108 mg, 20% (method B); pale beige oil; **5h**:**4h** = **2**:**1**. **For 5h** ^1H NMR (400 MHz, DMSO-d_6): δ 7.33 – 7.31 (m, 2H), 7.24 – 7.19 (m, 2H), 3.53 (d, J = 10.6 Hz, 1H), 3.33 – 3.29 (m, 2H), 3.01 – 2.96 (m, 1H), 2.83 – 2.77 (m, 1H), 2.68 – 2.57 (m, 1H), 2.46 (s, 3H), 2.26 – 2.23 (m, 1H), 1.96 (s, 3H) ppm; $^{13}C\{^1H\}$ NMR (100 MHz, DMSO-d_6): δ 208.4, 206.1, 140.5, 137.1, 128.0 (2C), 125.9 (2C), 65.9, 53.8, 46.1, 42.4, 38.9, 29.9, 14.7 ppm; HRMS (ESI$^+$) m/z: $[M+H]^+$ Calcd for $C_{15}H_{20}NO_2S^+$ 278.1209; found 278.1187. **For 4h** ^1H NMR (400 MHz, DMSO-d_6): δ 7.24 – 7.19 (m,

4H), 5.60 (s, 1H), 4.71 (br. s, 1H), 3.10 – 3.05 (m, 1H), 2.93 – 2.87 (m, 1H), 2.68 – 2.57 (m, 2H), 2.45 (s, 3H), 2.18 (s, 3H) ppm; $^{13}C\{^{1}H\}$ NMR (100 MHz, DMSO-*d*$_6$): *δ* 149.0, 147.5, 138.6, 136.2, 128.3 (2C), 125.9 (2C), 120.0, 104.8, 55.7, 41.4, 24.0, 14.9, 13.1 ppm; HRMS (ESI$^+$) m/z: [M+H]$^+$ Calcd for $C_{15}H_{18}NOS^+$ 260.1104; found 260.1107.

*2-Methyl-4-(2-methylphenyl)-4,5,6,7-tetrahydrofuro[3,2-c]pyridine (**4i**).* Yield: 263 mg, 58% (method A); yellow oil; ^{1}H NMR (400 MHz, DMSO-*d*$_6$): *δ* 7.17 – 7.08 (m, 4H), 5.54 (s, 1H), 4.96 (t, *J* = 2.0 Hz, 1H), 3.11 – 3.05 (m, 1H), 2.93 – 2.87 (m, 1H), 2.67 – 2.60 (m, 1H), 2.53 – 2.49 (m, 1H), 2.40 (s, 3H), 2.19 (s, 3H) ppm; $^{13}C\{^{1}H\}$ NMR (100 MHz, DMSO-*d*$_6$): *δ* 149.0, 147.9, 141.2, 136.0, 130.1, 127.7, 126.6, 125.2, 119.9, 104.8, 53.1, 41.5, 24.2, 18.7, 13.1 ppm; HRMS (ESI$^+$) m/z: [M+H]$^+$ Calcd for $C_{15}H_{18}NO^+$ 228.1383; found 228.1384.

*4-(3,4-Dimethoxyphenyl)-2-methyl-4,5,6,7-tetrahydrofuro[3,2-c]pyridine (**4j**).* Yield: 172 mg, 63% (method A); pale beige solid; mp = 108–109 °C (petroleum ether/ethyl acetate); ^{1}H NMR (400 MHz, DMSO-*d*$_6$): *δ* 6.91 (d, *J* = 1.9 Hz, 1H), 6.87 (d, *J* = 8.2 Hz, 1H), 6.78 (dd, *J* = 8.2, 1.9 Hz, 1H), 5.61 (s, 1H), 4.70 (br. s, 1H), 3.73 (s, 3H), 3.72 (s, 3H), 3.14 – 3.08 (m, 1H), 2.93 – 2.87 (m, 1H), 2.66 – 2.59 (m, 1H), 2.47 – 2.46 (m, 1H), 2.18 (s, 3H) ppm; $^{13}C\{^{1}H\}$ NMR (100 MHz, DMSO-*d*$_6$): *δ* 148.9, 148.5, 147.9, 147.3, 136.0, 120.3, 119.7, 111.9, 111.5, 104.9, 55.9, 55.5, 55.4, 41.5, 24.0, 13.1 ppm; HRMS (ESI$^+$) m/z: [M+H]$^+$ Calcd for $C_{16}H_{20}NO_3^+$ 274.1438; found 274.1445.

*2-Methyl-4-(3-phenoxyphenyl)-4,5,6,7-tetrahydrofuro[3,2-c]pyridine (**4k**).* Yield: 143 mg, 47% (method A); yellow oil; ^{1}H NMR (400 MHz, DMSO-*d*$_6$): *δ* 7.40 – 7.30 (m, 4H), 7.15 – 7.10 (m, 2H), 7.00 – 6.98 (m, 2H), 6.88 (dd, *J* = 8.0, 1.9 Hz, 1H), 5.65 (s, 1H), 4.75 (br. s, 1H), 3.10 – 3.05 (m, 1H), 2.94 – 2.88 (m, 1H), 2.64 – 2.57 (m, 1H), 2.51 –

2.47 (m, 1H), 2.18 (s, 3H) ppm; $^{13}C\{^1H\}$ NMR (100 MHz, DMSO-d_6): δ 156.6, 156.3, 149.1, 147.5, 146.0, 129.8 (2C), 129.4, 123.2, 122.9, 119.8, 118.3 (2C), 118.0, 117.0, 104.8, 55.8, 41.4, 24.0, 13.1 ppm; HRMS (ESI$^+$) m/z: $[M+H]^+$ Calcd for $C_{20}H_{20}NO_2^+$ 306.1489; found 306.1486.

*2-Methyl-4-(thiophen-2-yl)-4,5,6,7-tetrahydrofuro[3,2-c]pyridine (**4l**).* Yield: 66 mg, 15% (method B); yellow oil; ^1H NMR (400 MHz, DMSO-d_6): δ 7.39 (dd, J = 4.7, 1.0 Hz, 1H), 7.00 – 6.98 (m, 2H), 5.87 (s, 1H), 5.08 (br. s, 1H), 3.12 – 3.06 (m, 1H), 3.02 – 2.96 (m, 1H), 2.59 – 2.55 (m, 2H), 2.25 (s, 3H) ppm; $^{13}C\{^1H\}$ NMR (100 MHz, DMSO-d_6): δ 149.0, 148.7, 147.3, 126.2, 124.3, 124.2, 120.1, 104.9, 51.3, 40.6, 23.9, 13.1 ppm; HRMS (ESI$^+$) m/z: $[M+H]^+$ Calcd for $C_{12}H_{14}NOS^+$ 220.0791; found 220.0786.

3. Synthesis of 3-(2-oxopropyl)-2-phenylpiperidin-4-one (5a). To a solution of 2-methyl-4-phenyl-4,5,6,7-tetrahydrofuro[3,2-*c*]pyridine (**4a**, 0.8 mmol, 176 mg) in 1,4-dioxane (2 mL) was added portionwise 30% HCl (2.5 mL). The reaction mixture was refluxed for 9 h (TLC control). The yield of **5a** (70%) was determined by GC/MS using an internal standard.

4. Synthesis of 1-(4-chlorophenyl)-2-methyl-4-phenyl-4,5,6,7-tetrahydro-1*H*-pyrrolo[3,2-*c*]pyridine (6a). To a solution of 2-methyl-4-phenyl-4,5,6,7-tetrahydrofuro[3,2-*c*]pyridine (**4a**, 0.8 mmol, 176 mg) in 1,4-dioxane (2 mL) was added portionwise 30% HCl (2.5 mL). The reaction mixture was refluxed for 9 h (TLC control). Then 4-chloroaniline (0.8 mmol, 105 mg) and 30% HCl (1.8 mL) were added. The reaction mixture was refluxed for 4 h (TLC control). Then aq. saturated solution of $NaHCO_3$ was added and the mixture was stirred overnight at room temperature. The formed precipitate was filtered and the filtrate was extracted with ethyl acetate (3 ×

10 mL). The combined organic layers were washed with brine (3 × 10 mL), dried with anhydrous Na_2SO_4 and concentrated to dryness. The residue and precipitate were combined. The product was purified by column chromatography on silica gel using a mixture of petroleum ether/ethyl acetate 3:1 as an eluent. Yield: 51 mg, 19%; yellow oil; ^{1}H NMR (400 MHz, DMSO-*d₆*): δ 7.55 (d, J = 7.6 Hz, 2H), 7.38 (d, J = 7.6 Hz, 2H), 7.34 – 7.29 (m, 4H), 7.24 – 7.21 (m, 1H), 5.44 (s, 1H), 4.83 (s, 1H), 3.09 – 3.06 (m, 1H), 2.95 (s, 1H), 2.90 – 2.83 (m, 1H), 2.79 (s, 1H), 2.23 – 2.18 (m, 1H), 1.98 (s, 3H) ppm; ^{13}C{^{1}H} NMR (100 MHz, DMSO-d_6): δ 145.0, 136.7, 131.6, 129.1 (2C), 128.9 (2C), 127.8 (2C), 127.7 (2C), 126.6, 126.5, 126.1, 119.6, 105.2, 57.3, 42.1, 23.6, 12.3 ppm; HRMS (ESI$^+$) m/z: [M+H]$^+$ Calcd for $C_{20}H_{20}ClN_2^+$ 323.1310; found 323.1312.

5. Synthesis of 2,5-dimethyl-4-phenyl-4,5,6,7-tetrahydrofuro[3,2-*c*]pyridine (4r). In a manner similar as described earlier [3]. To a stirred solution of 2-methyl-4-phenyl-4,5,6,7-tetrahydrofuro[3,2-*c*]pyridine (**4a**, 0.7 mmol, 145 mg) in dry THF (2 mL) sodium hydride (3.6 mmol, 60% dispersion in mineral oil, 87 mg) was added portionwise. The reaction mixture was stirred at room temperature for 5 min followed by the addition of methyl iodide (1.2 mmol, 76 μL). The reaction mixture was stirred for 3 h at ambient temperature (TLC control) and then poured into H_2O (50 mL). The product was extracted with ethyl acetate (3 × 30 mL). The combined organic layers were washed with brine (3 × 20 mL), dried with anhydrous Na_2SO_4 and concentrated to dryness under reduced pressure. The product was purified by column chromatography on silica gel using a mixture of petroleum ether/ethyl acetate 3:1 as an eluent. Yield: 69 mg, 45%; yellow oil; ^{1}H NMR (400 MHz, DMSO-d_6): δ 7.33 – 7.22 (m, 5H), 5.36 (d, J = 0.8 Hz, 1H), 3.99 (t, J = 2.0 Hz, 1H), 3.08 – 3.03 (m, 1H), 2.87 – 2.79 (m, 1H), 2.64 – 2.56 (m, 2H), 2.14 (s, 3H), 2.12 (s, 3H) ppm; ^{13}C{^{1}H} NMR (100 MHz, DMSO-d_6): δ 149.7, 145.9, 142.5, 128.0 (2C), 127.9 (2C), 127.0, 120.4, 105.0,

65.2, 51.4, 42.5, 23.3, 13.1 ppm; HRMS (ESI$^+$) m/z: $[M+H]^+$ Calcd for $C_{15}H_{18}NO^+$ 228.1383; found 228.1387.

6. Synthesis of 1-(2-methyl-4-phenyl-6,7-dihydrofuro[3,2-*c*]pyridin-5(4*H*)-yl)ethan-1-one (4q). In a manner similar as described earlier [3]. To a solution of 2-methyl-4-phenyl-4,5,6,7-tetrahydrofuro[3,2-*c*]pyridine (**4a**, 0.8 mmol, 176 mg), TEA (2.5 mmol, 347 μL) in dry acetonitrile (2 mL) and acetyl chloride (2.5 mmol, 178 μL) were added dropwise. The reaction mixture was refluxed for 1 h (TLC control). Then the reaction mixture was poured into aq. NaOH (0.1 M, 50 mL) and the product was extracted with ethyl acetate (3 × 10 mL). The combined organic layers were washed with brine (3 × 10 mL), dried with anhydrous Na_2SO_4 and concentrated to dryness under reduced pressure. The product was purified by column chromatography on silica gel using a mixture of petroleum ether/ethyl acetate 5:1 as an eluent. Yield: 160 mg, 75%; yellow oil; ^{1}H NMR (400 MHz, DMSO-d_6): δ 7.32 – 7.26 (m, 5H), 6.50 (s, 1H), 5.99 (s, 1H), 3.95 – 3.90 (m, 1H), 3.19 – 3.12 (m, 1H), 2.87 – 2.80 (m, 1H), 2.63 – 2.60 (m, 1H), 2.25 (s, 3H), 2.12 (s, 3H) ppm; ^{13}C{^{1}H} NMR (100 MHz, DMSO-d_6): δ 169.1, 151.1, 147.7, 141.2, 128.7 (2C), 128.0 (2C), 127.8, 117.8, 106.0, 50.8, 40.1, 24.3, 22.0, 13.7 ppm; HRMS (ESI$^+$) m/z: $[M+H]^+$ Calcd for $C_{16}H_{18}NO_2^+$ 256.1332; found 256.1340.

7. Copies of ^{1}H, ^{13}C NMR spectra of new compounds

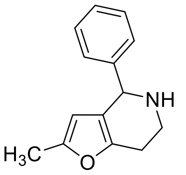

4a

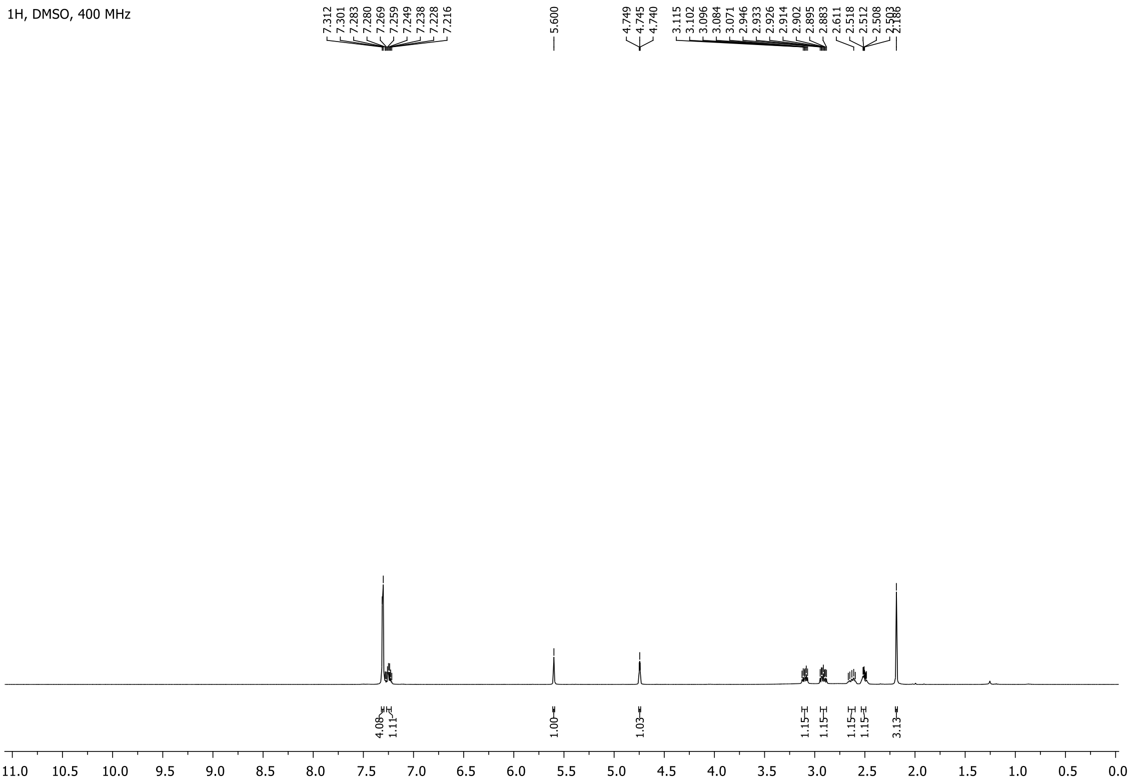

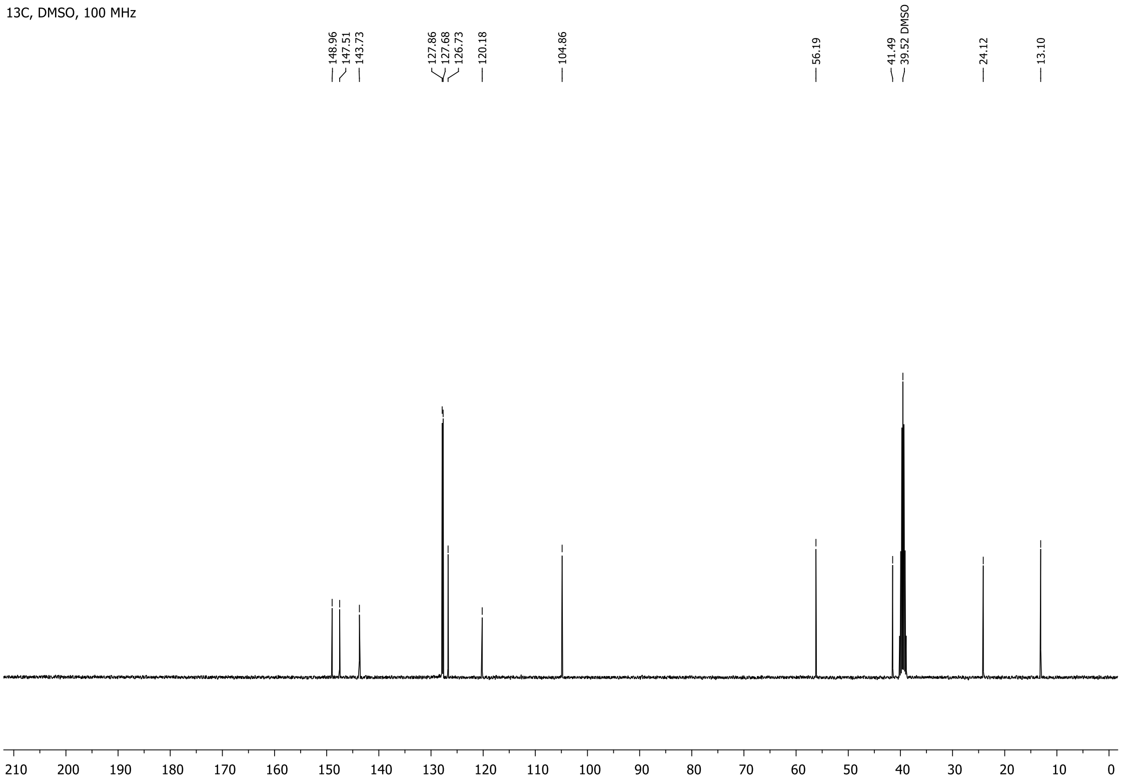

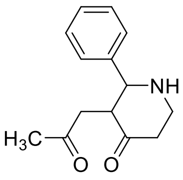

5a

1H, DMSO, 400 MHz

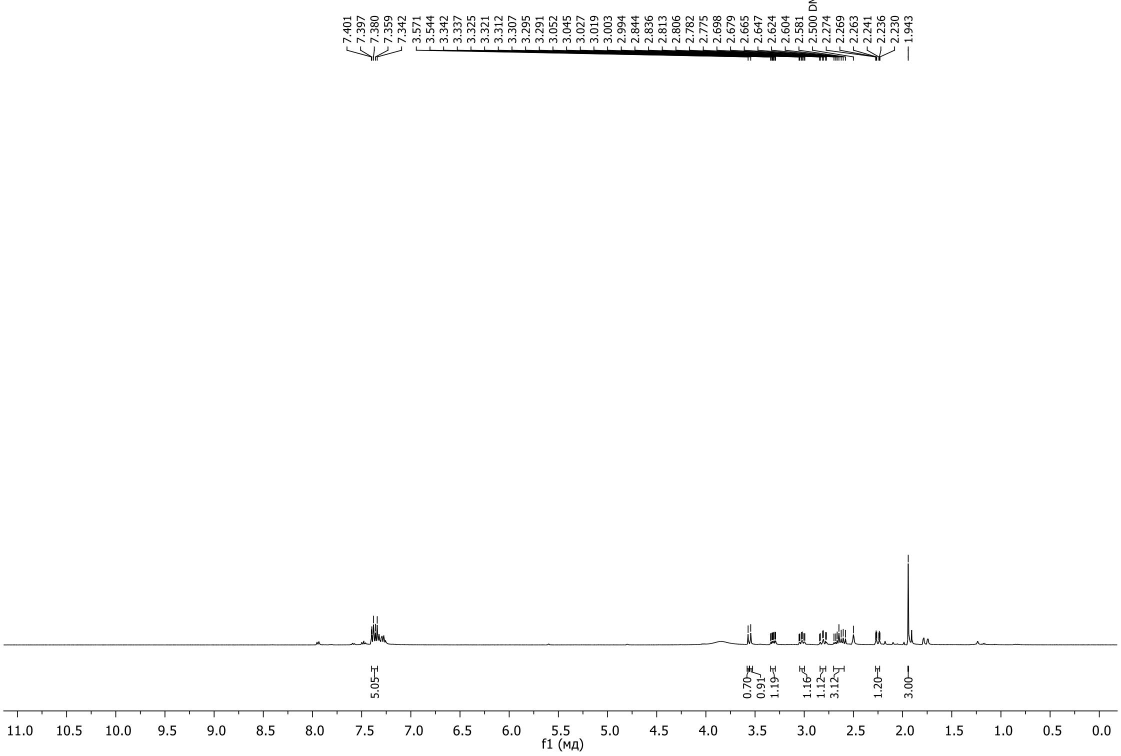

13C, DMSO, 100 MHz

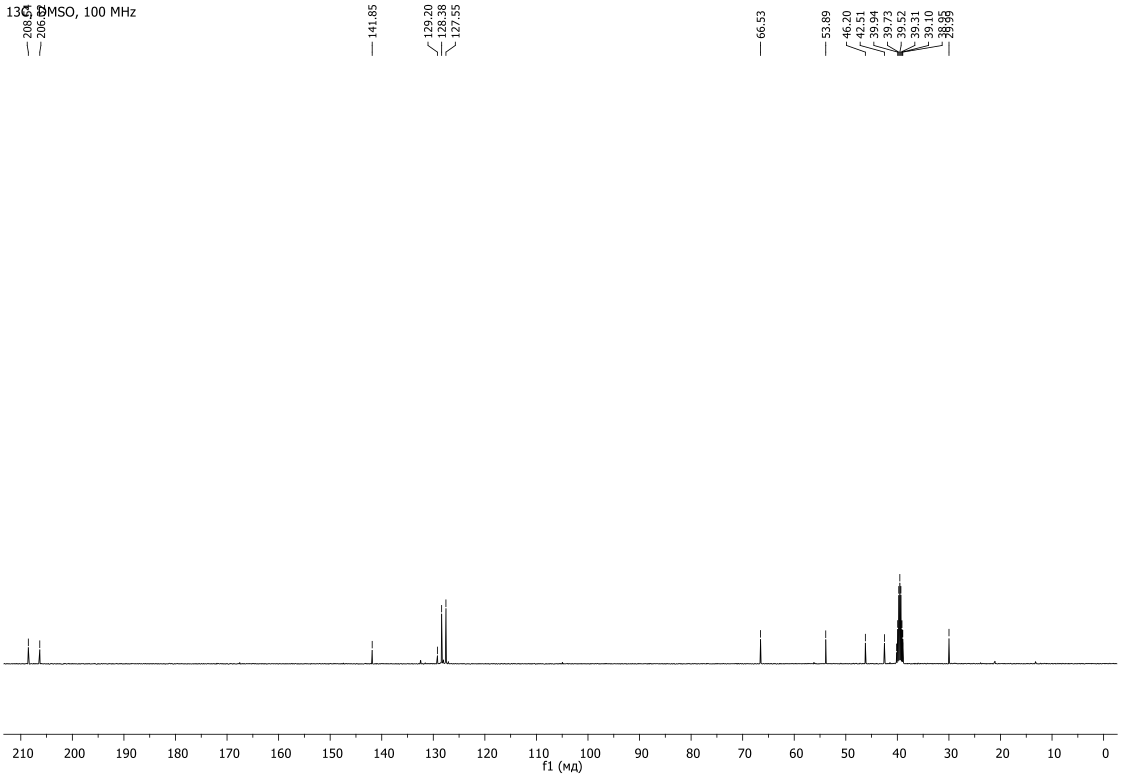

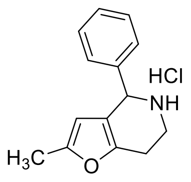

4a′

1H, DMSO, 400 MHz

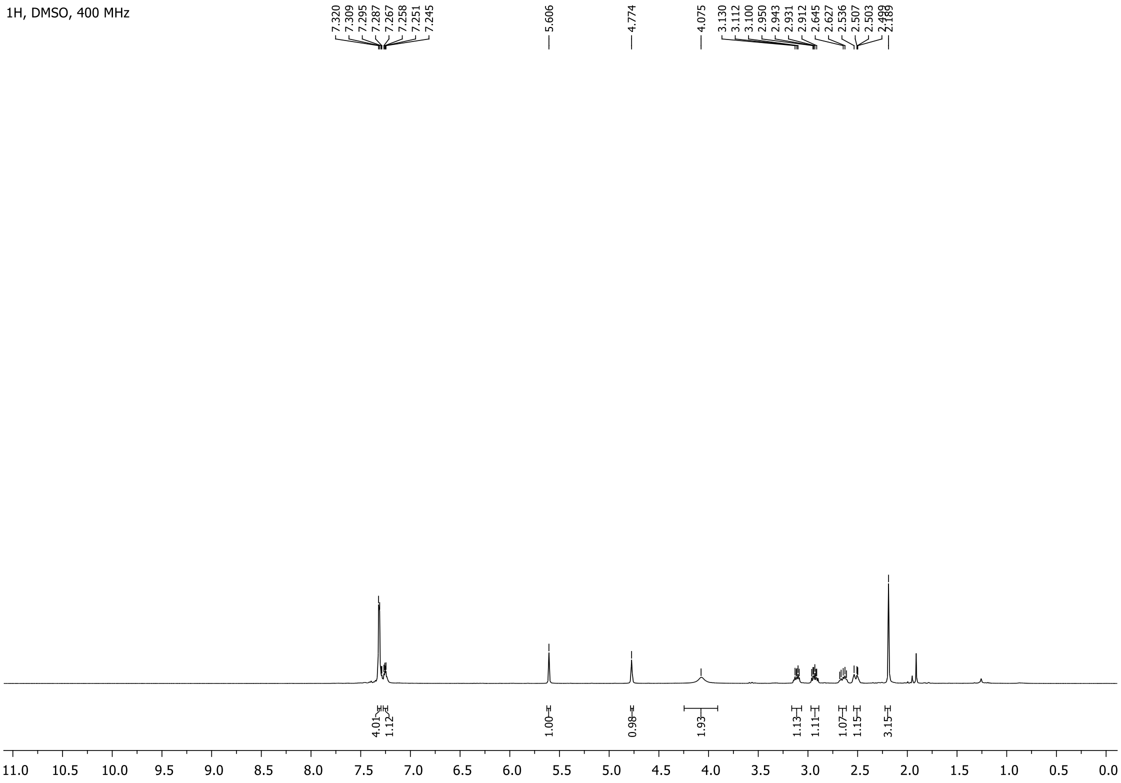

13C, DMSO, 100 MHz

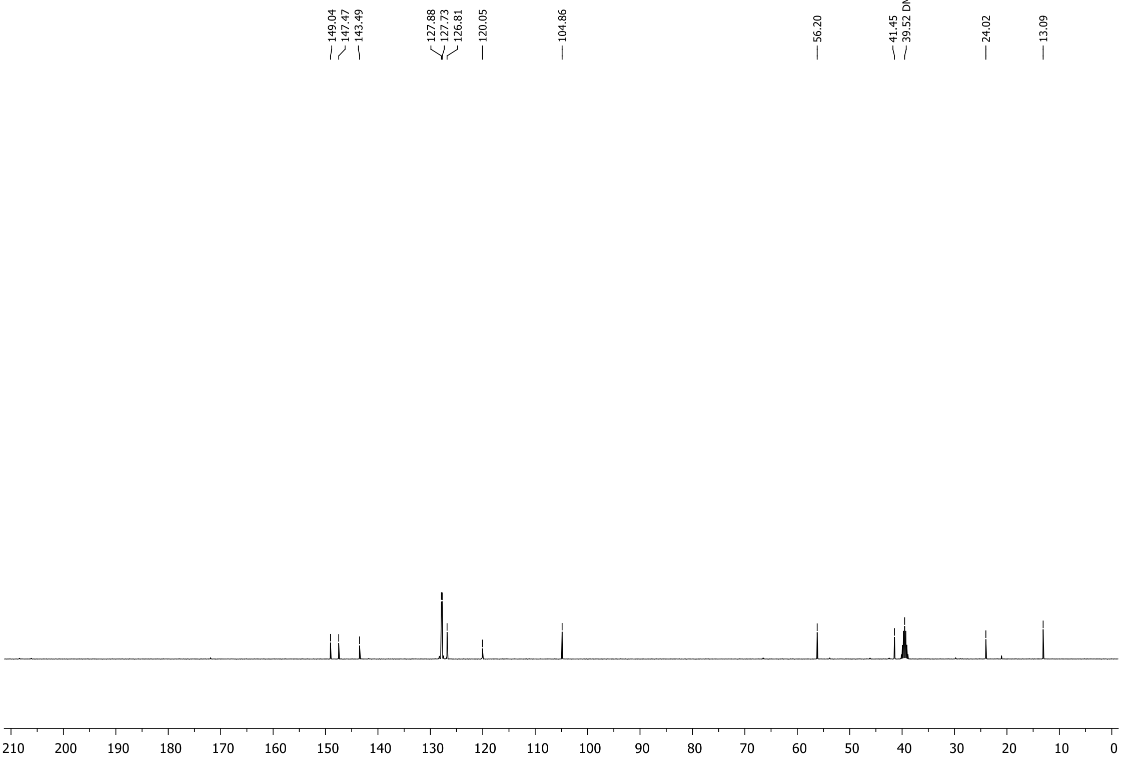

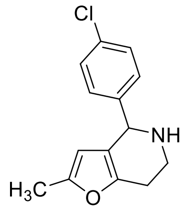

Cl
NH
H_3C
O

4b

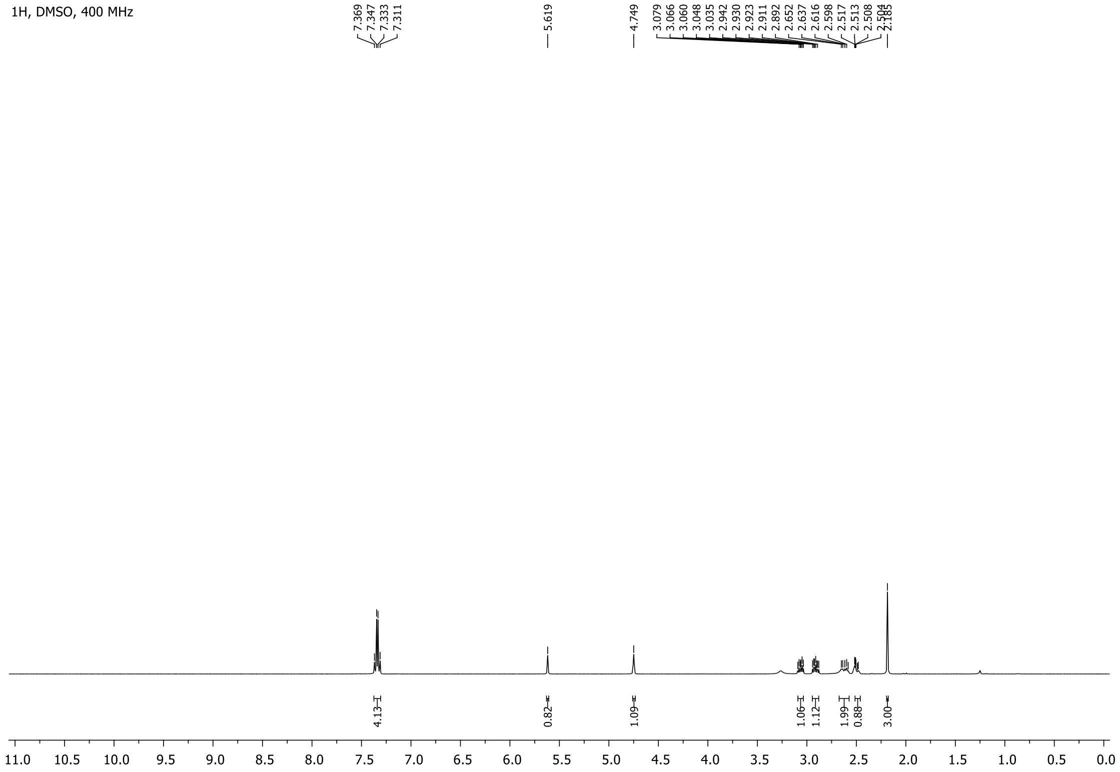

1H, DMSO, 400 MHz
7.369
7.347
7.333
7.311
5.619
4.749
3.079
3.066
3.060
3.048
3.035
2.942
2.930
2.923
2.911
2.892
2.652
2.637
2.616
2.598
2.517
2.513
2.508
2.504
2.185
4.13
0.82
1.09
1.06
1.12
1.99
0.88
3.00
11.0 10.5 10.0 9.5 9.0 8.5 8.0 7.5 7.0 6.5 6.0 5.5 5.0 4.5 4.0 3.5 3.0 2.5 2.0 1.5 1.0 0.5 0.0

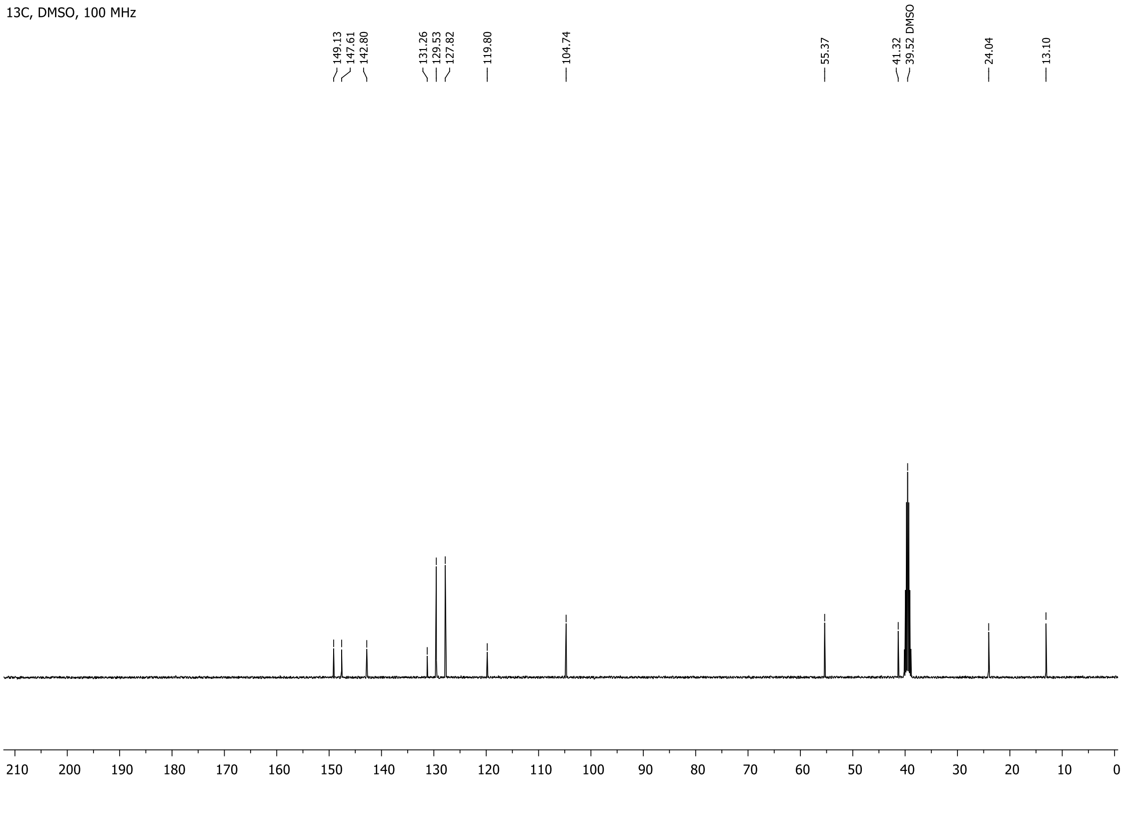

13C, DMSO, 100 MHz
149.13
147.61
142.80
131.26
129.53
127.82
119.80
104.74
55.37
41.32
39.52 DMSO
24.04
13.10
210 200 190 180 170 160 150 140 130 120 110 100 90 80 70 60 50 40 30 20 10 0

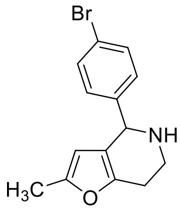
Br
NH
H3C
O

4c

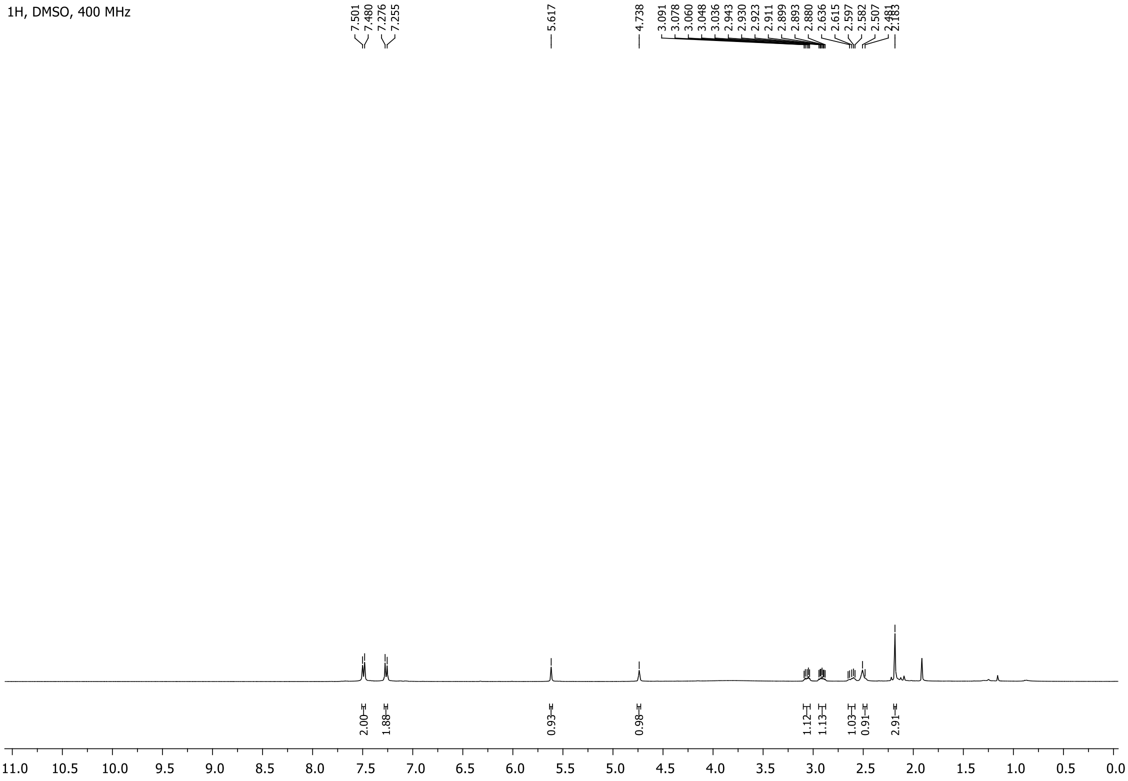
1H, DMSO, 400 MHz
7.501
7.480
7.276
7.255
5.617
4.738
3.091
3.078
3.060
3.048
3.036
2.943
2.930
2.923
2.911
2.899
2.893
2.880
2.636
2.615
2.597
2.582
2.507
2.481
2.183
2.00
1.88
0.93
0.98
1.12
1.13
1.03
0.91
2.91
11.0 10.5 10.0 9.5 9.0 8.5 8.0 7.5 7.0 6.5 6.0 5.5 5.0 4.5 4.0 3.5 3.0 2.5 2.0 1.5 1.0 0.5 0.0

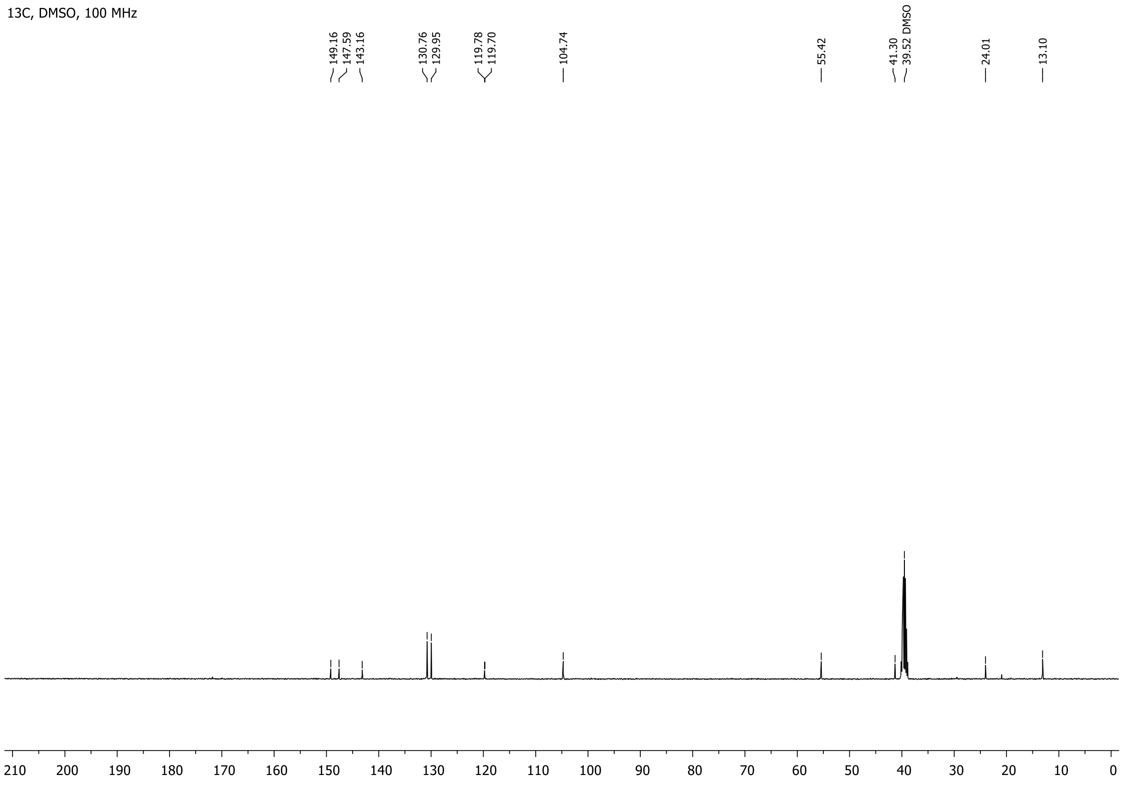
13C, DMSO, 100 MHz
149.16
147.59
143.16
130.76
129.95
119.78
119.70
104.74
55.42
41.30
39.52 DMSO
24.01
13.10
210 200 190 180 170 160 150 140 130 120 110 100 90 80 70 60 50 40 30 20 10 0

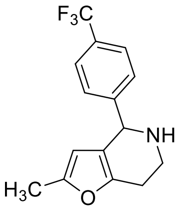

4d

1H, DMSO, 400 MHz

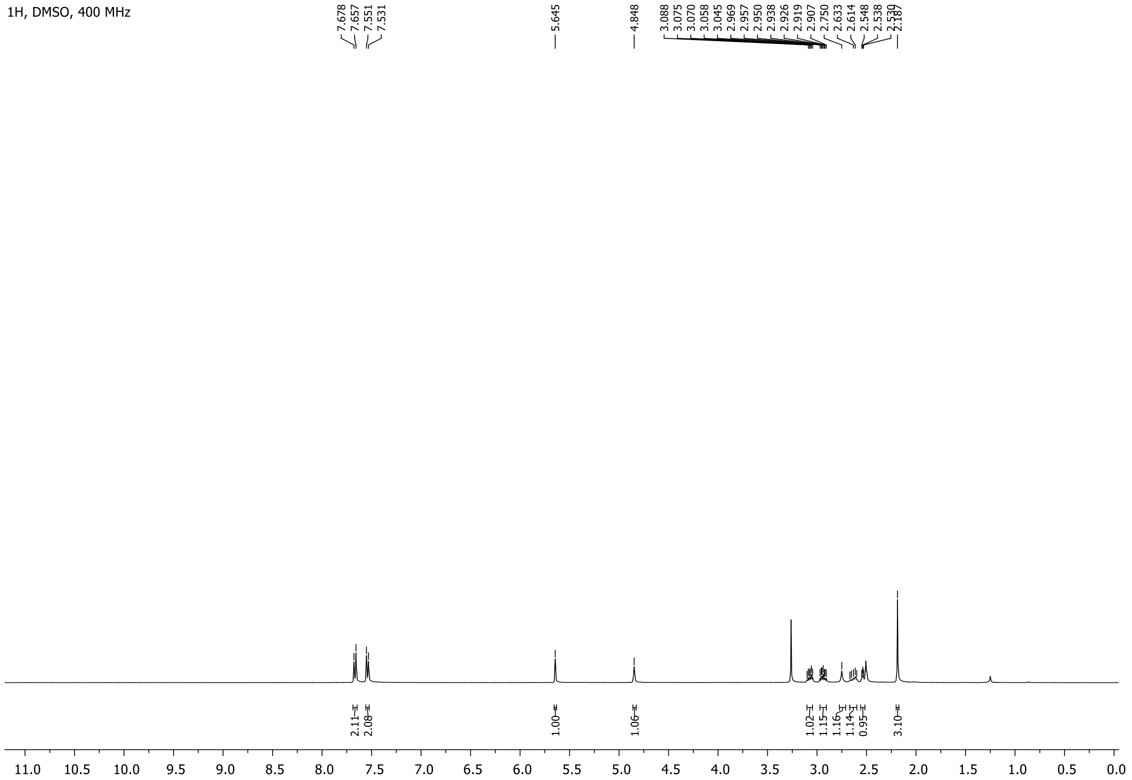

13C, DMSO, 100 MHz

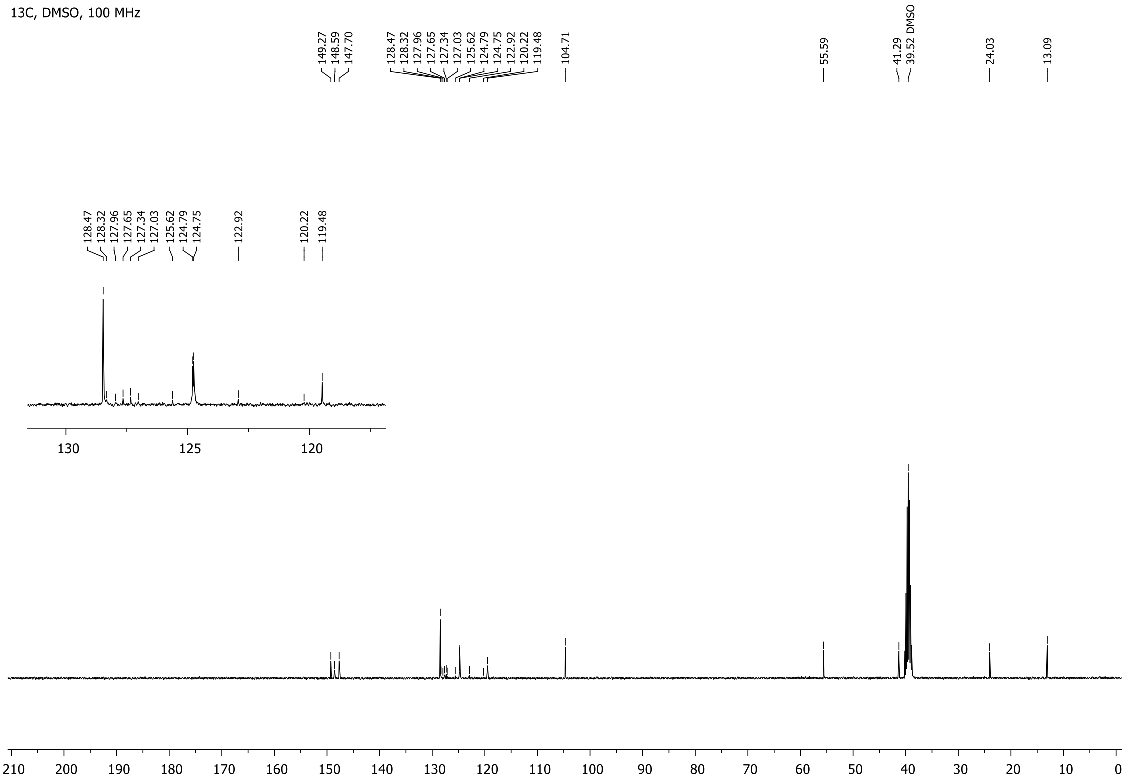

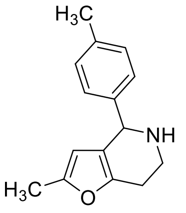

4e

1H, DMSO, 400 MHz

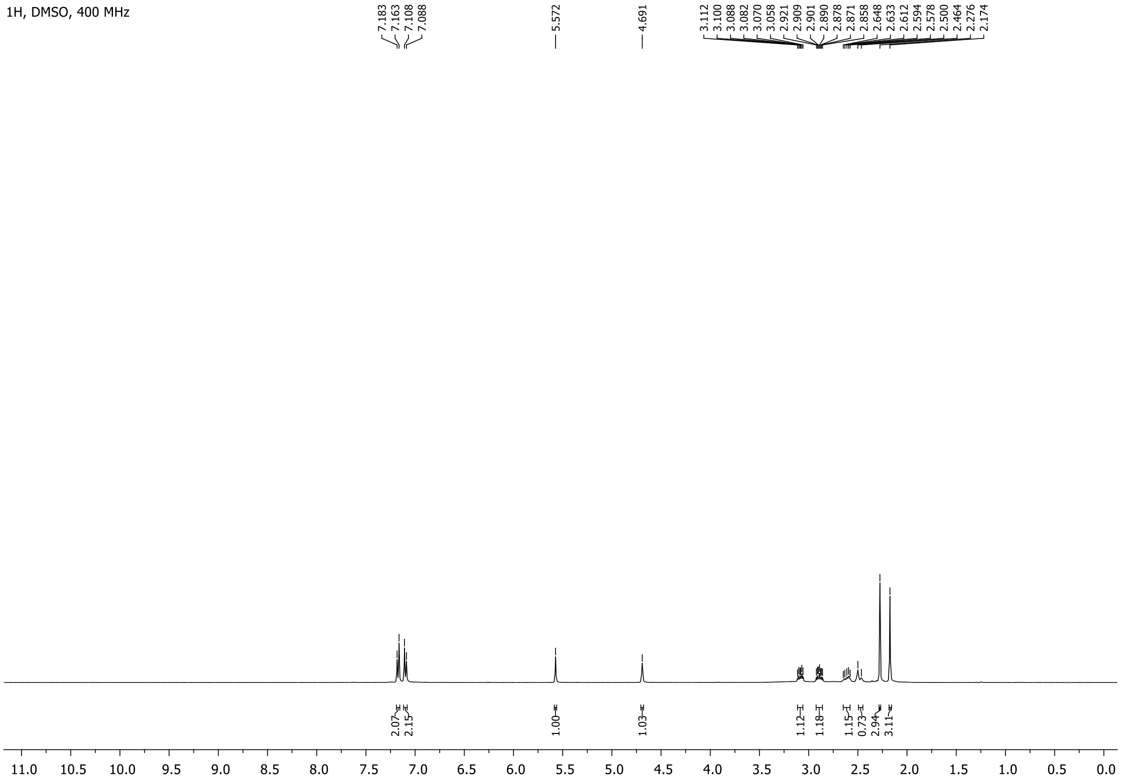

13C, DMSO, 100 MHz

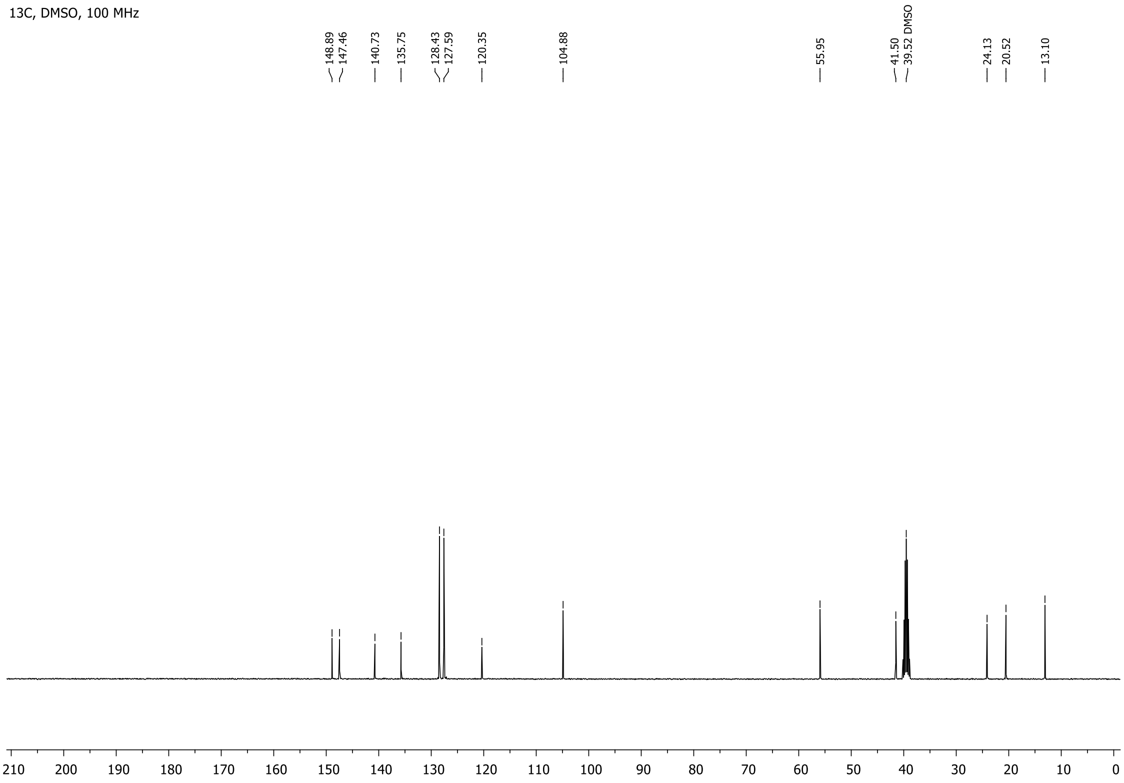

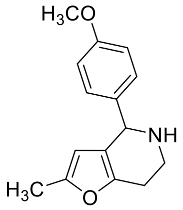

4f

1H, DMSO, 400 MHz

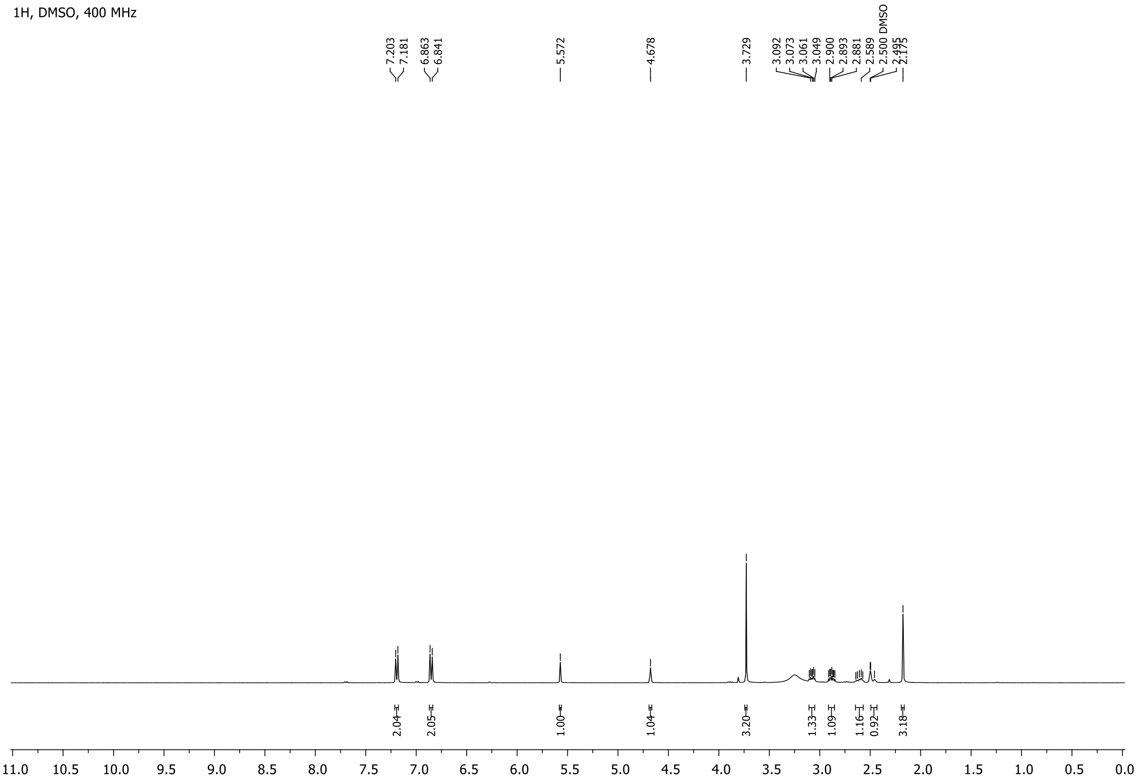

13C, DMSO, 100 MHz

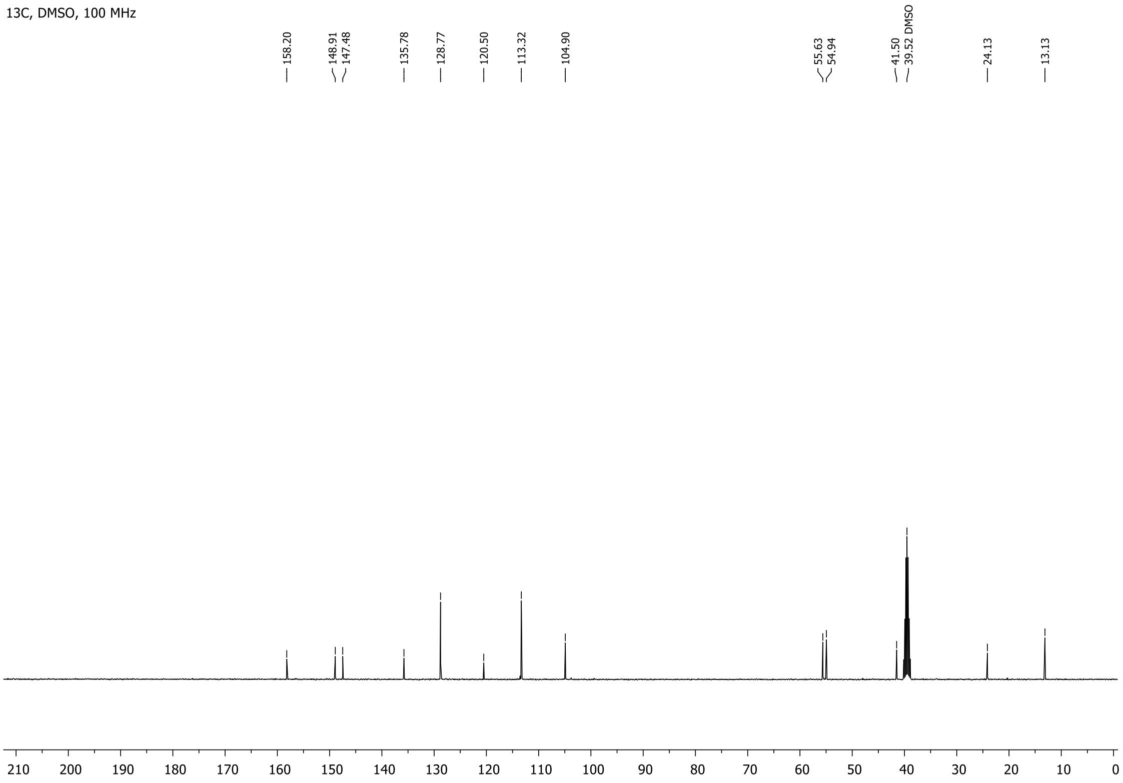

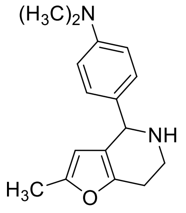

4g

1H, DMSO, 400 MHz

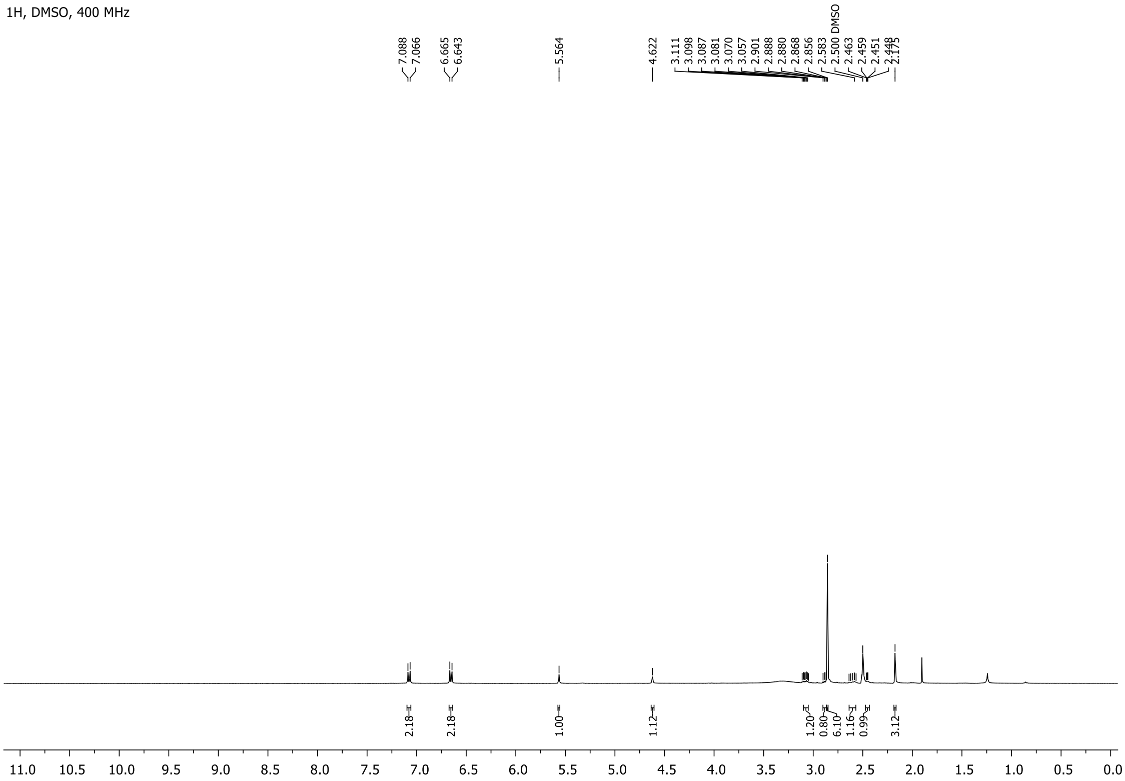

13C, DMSO, 100 MHz

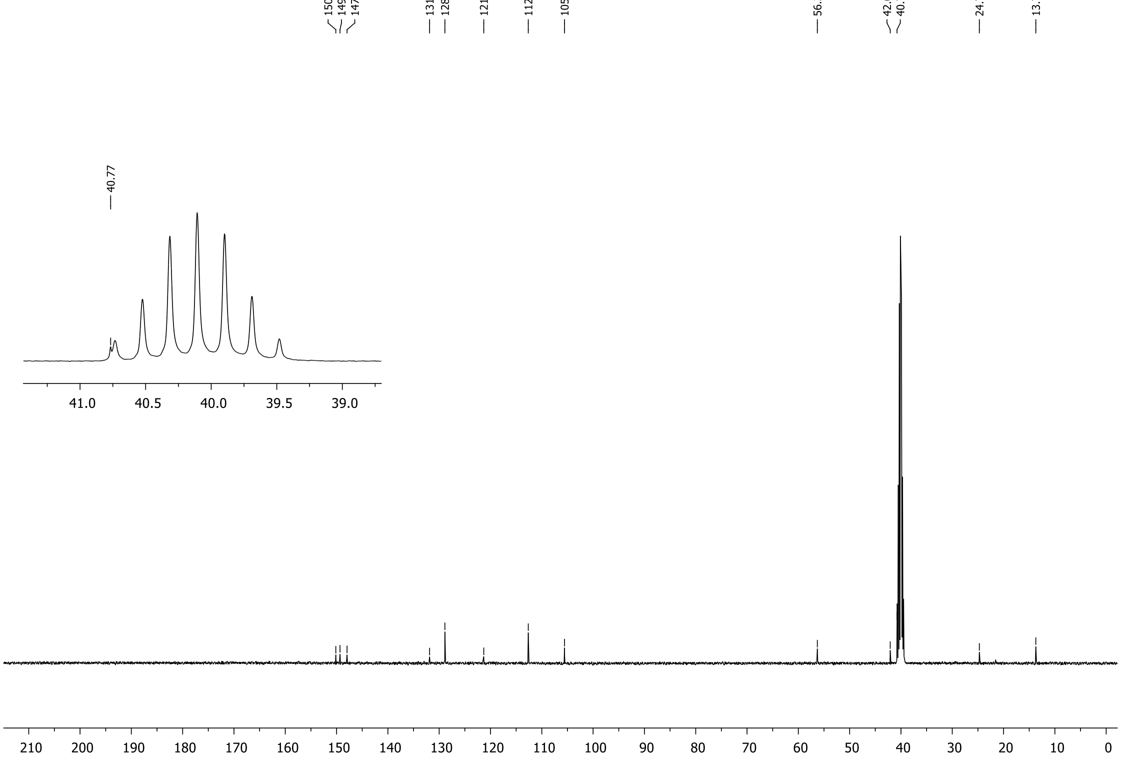

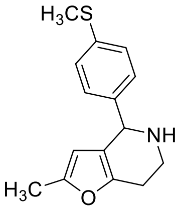

4h

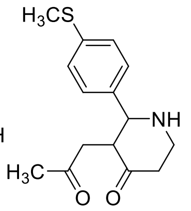

5h

1H, DMSO, 400 MHz

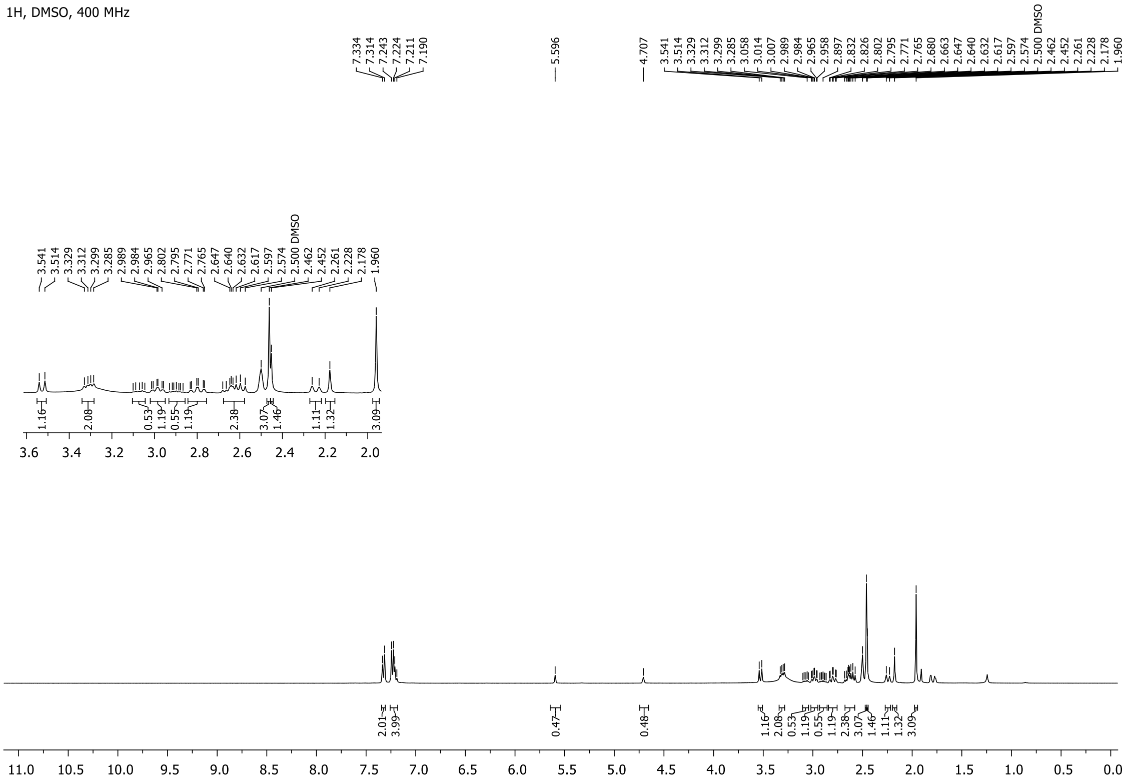

13C, DMSO, 100 MHz

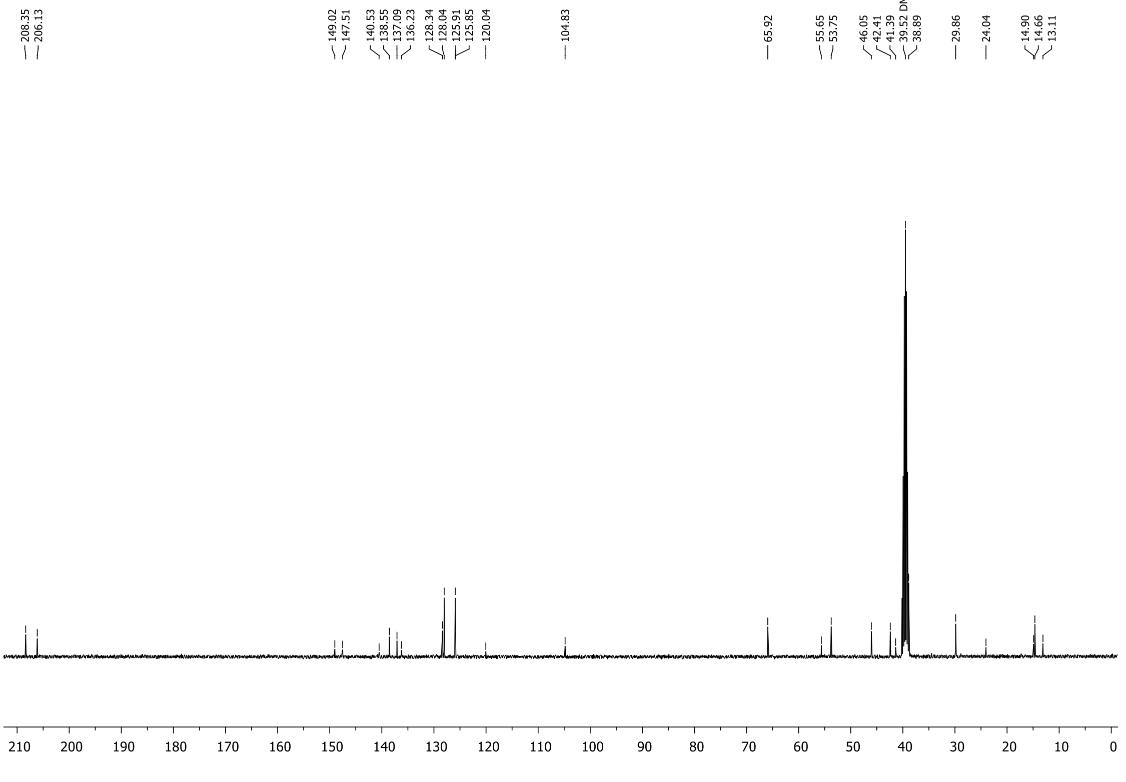

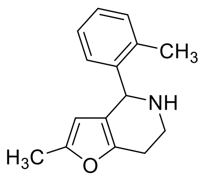

4i

1H, DMSO, 400 MHz

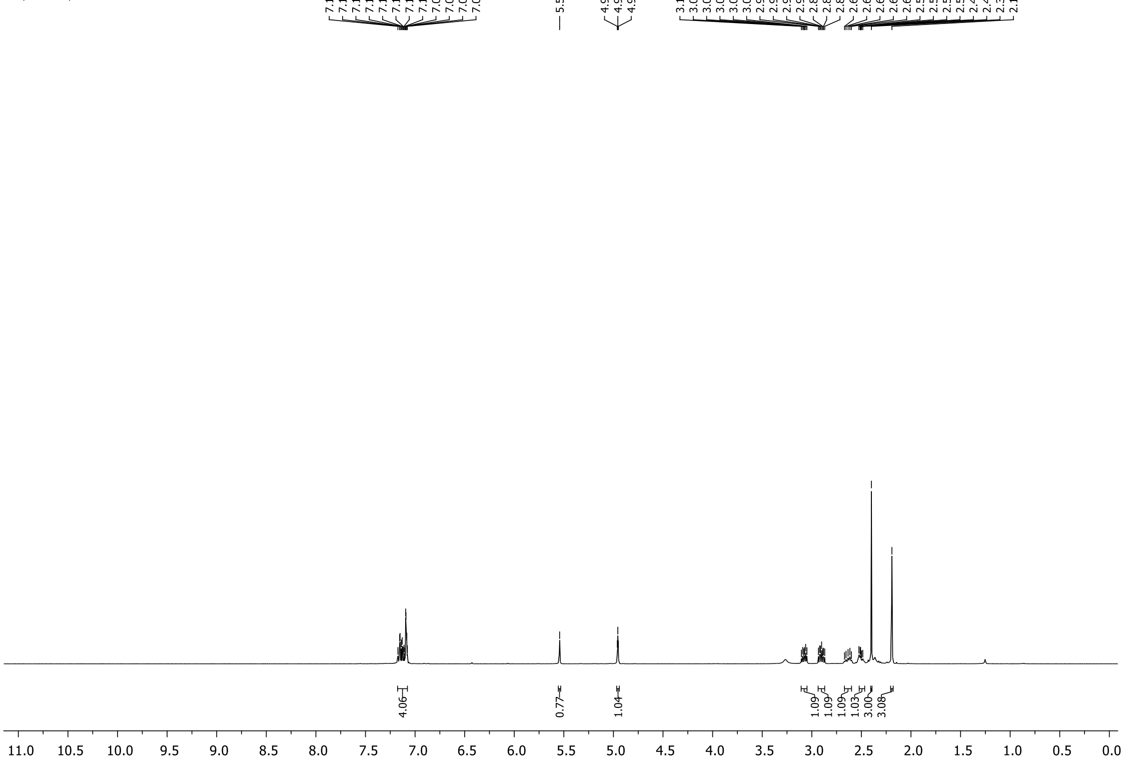

13C, DMSO, 100 MHz

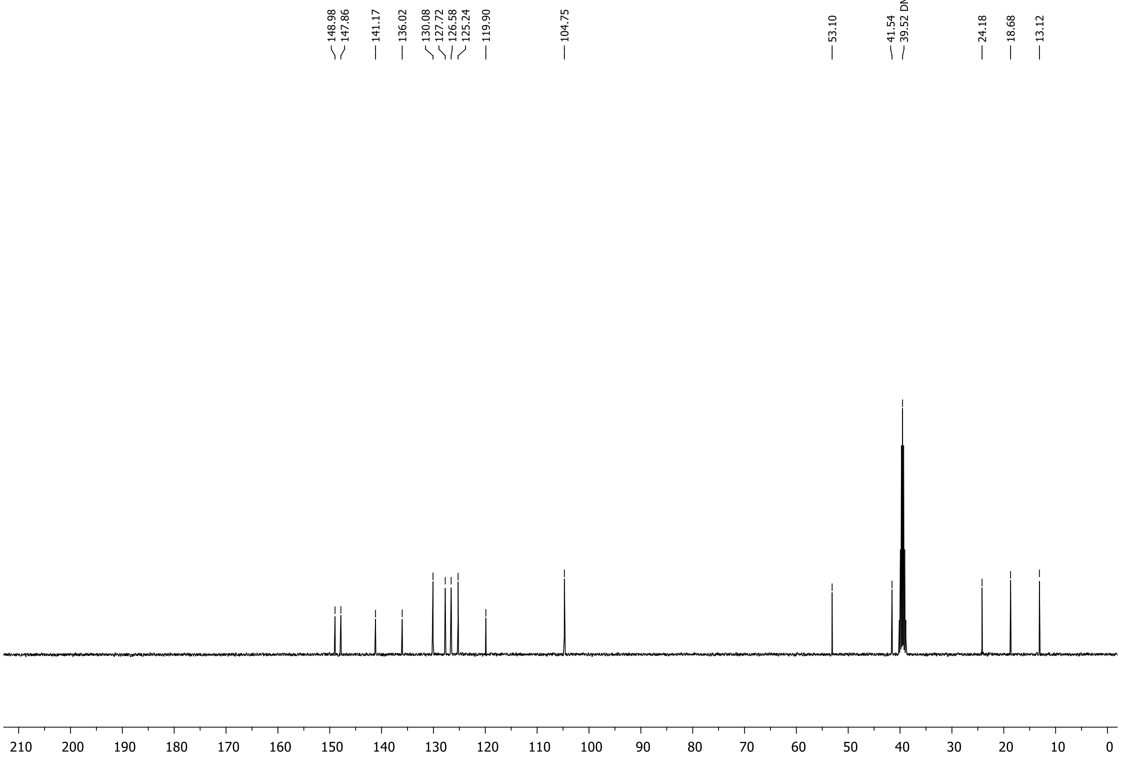

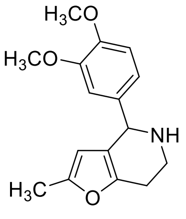

4j

1H, DMSO, 400 MHz

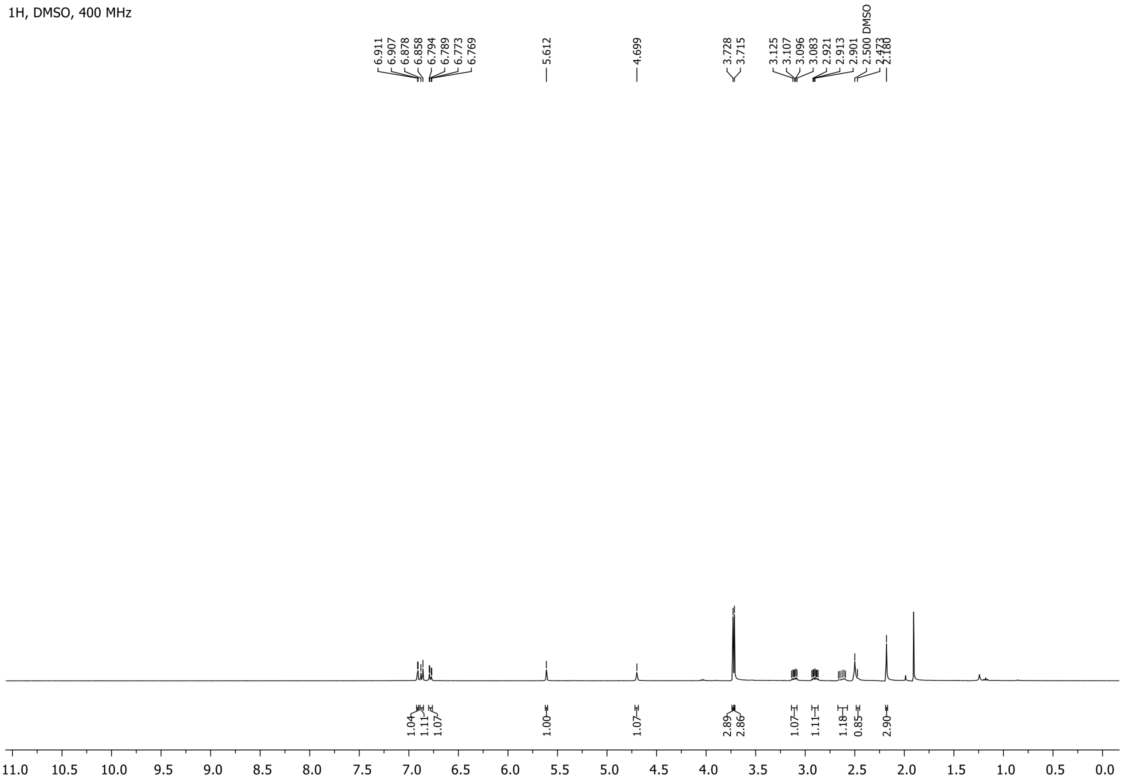

13C, DMSO, 100 MHz

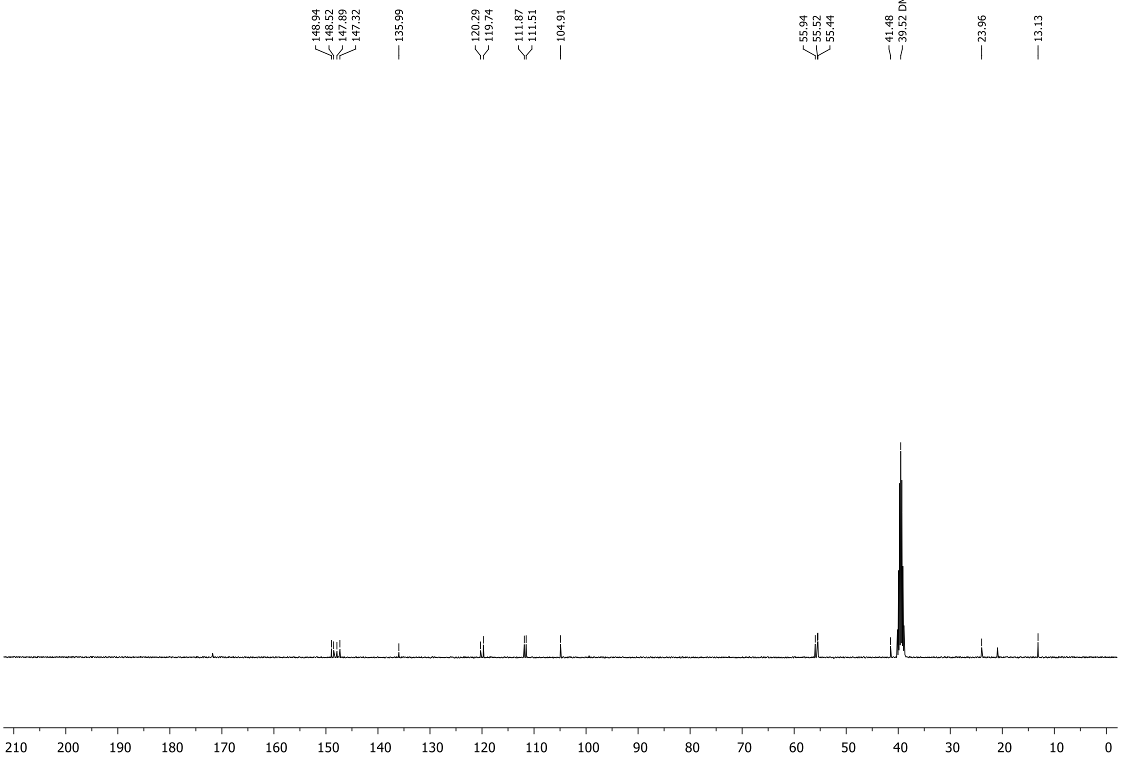

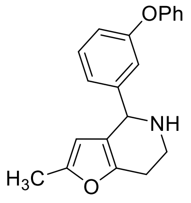

4k

1H, DMSO, 400 MHz

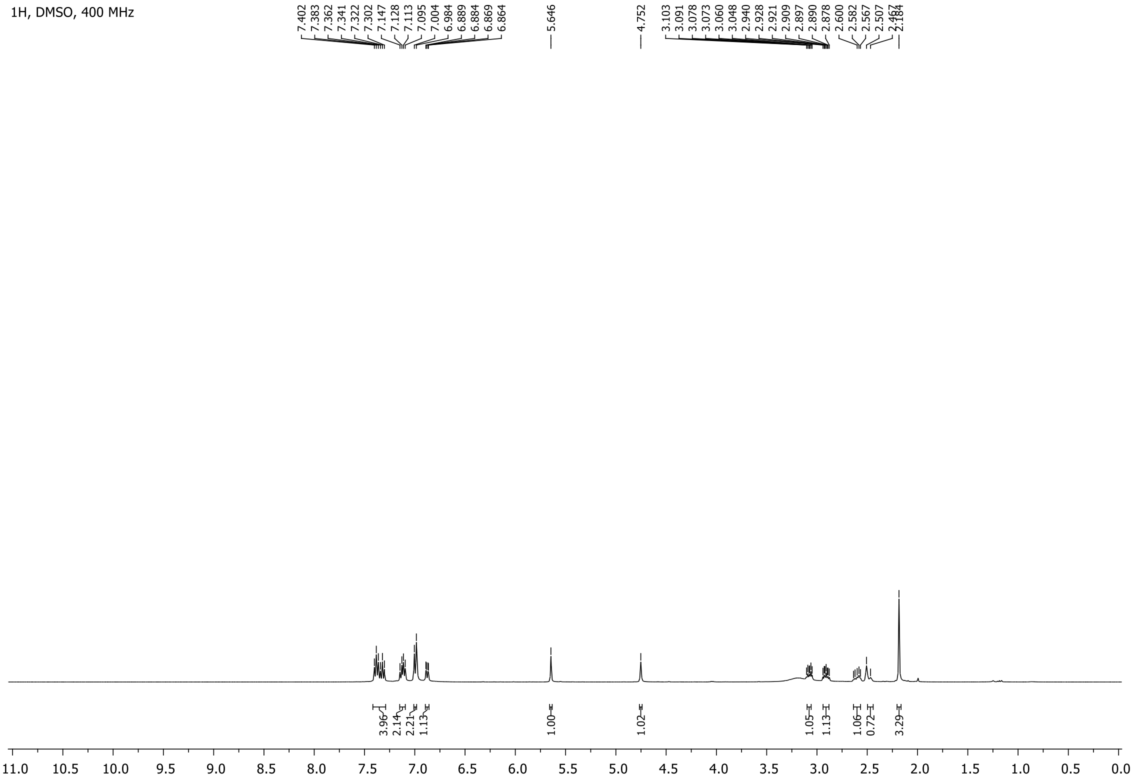

13C, DMSO, 100 MHz

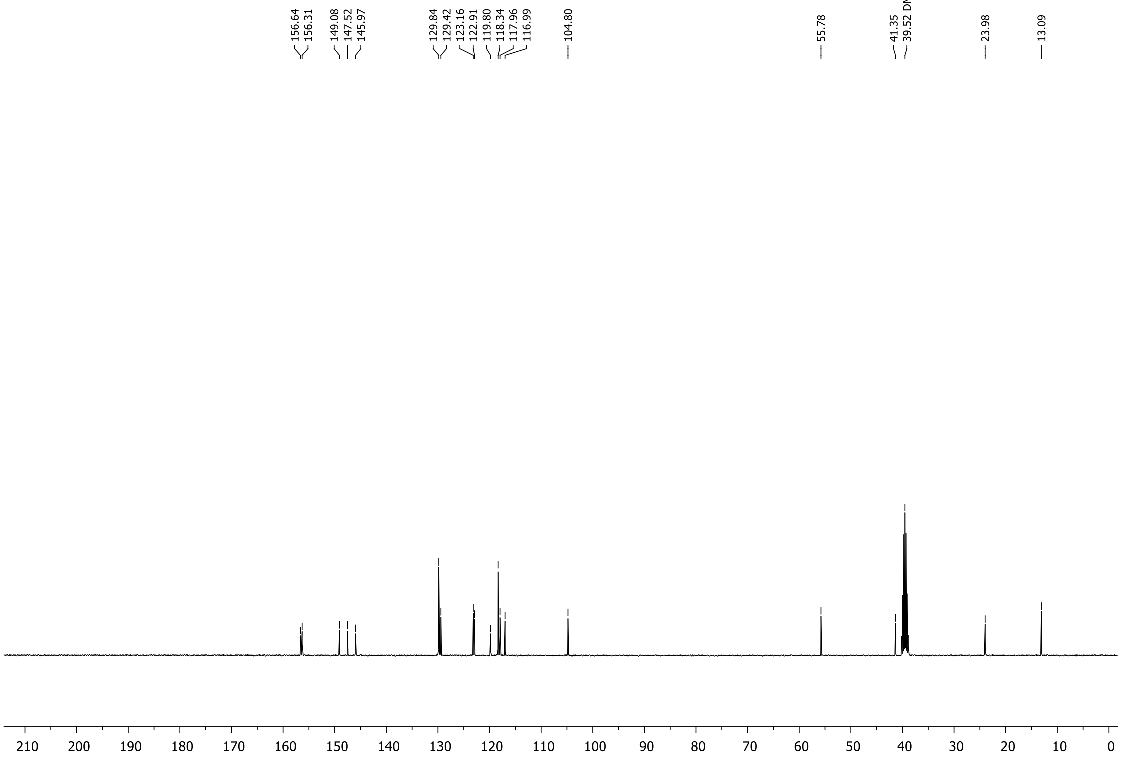

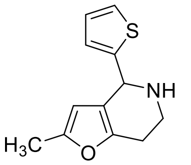

4l

1H, DMSO, 400 MHz

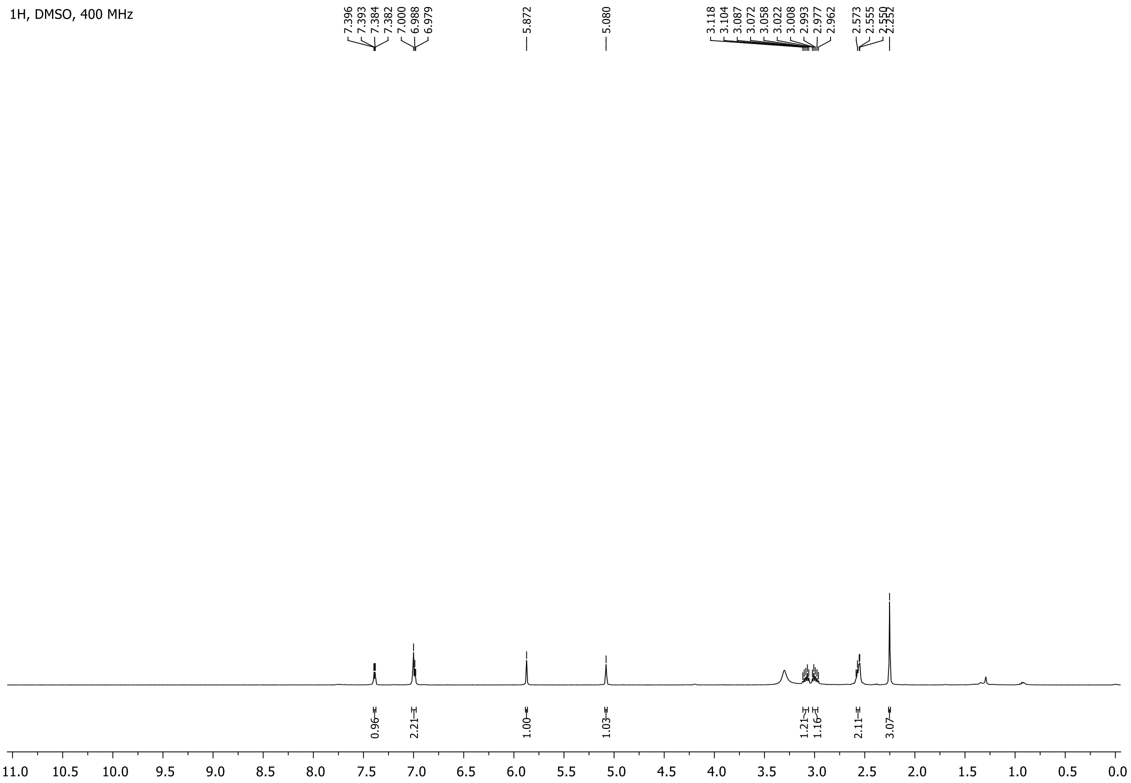

13C, DMSO, 100 MHz

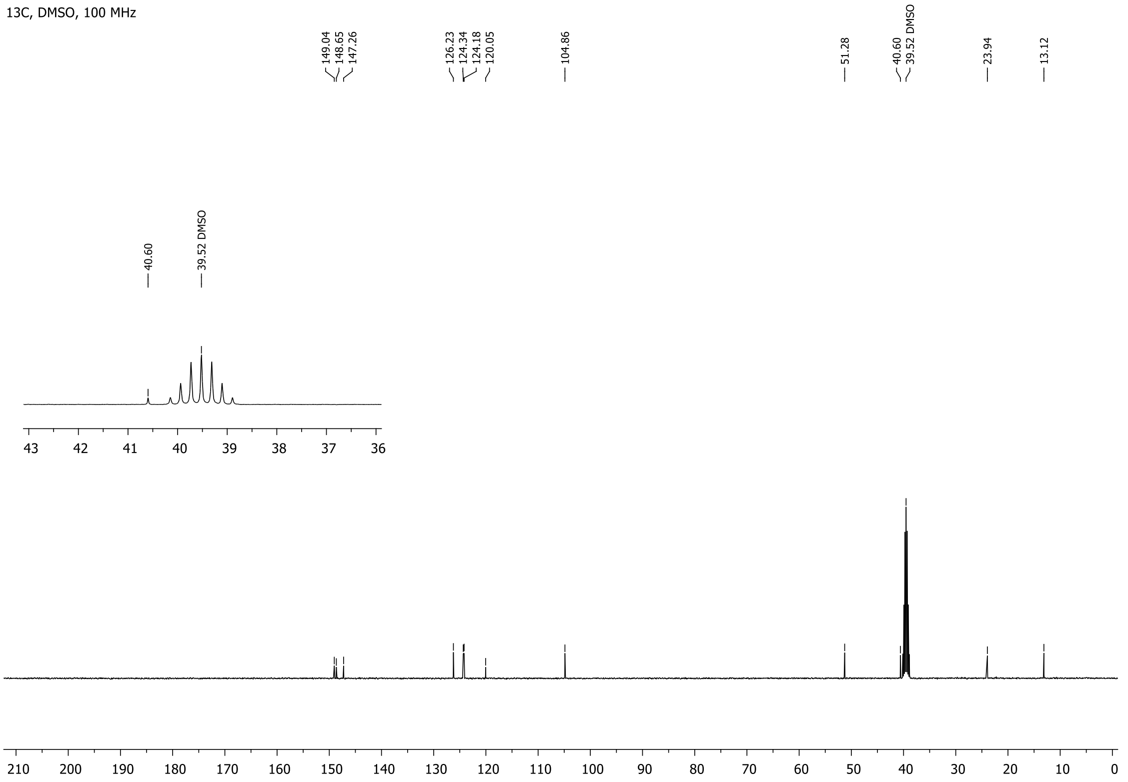

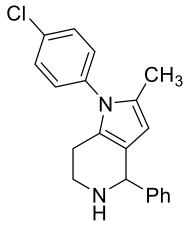

6a

1H, DMSO, 400 MHz

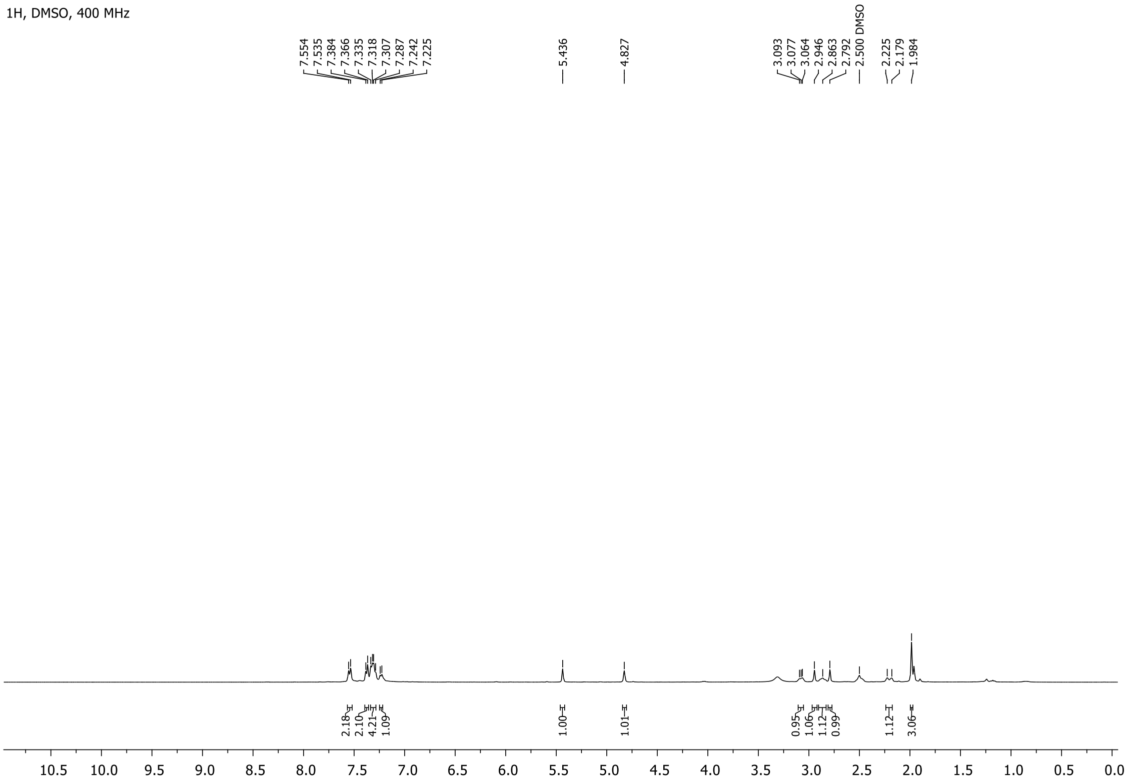

13C, DMSO, 100 MHz

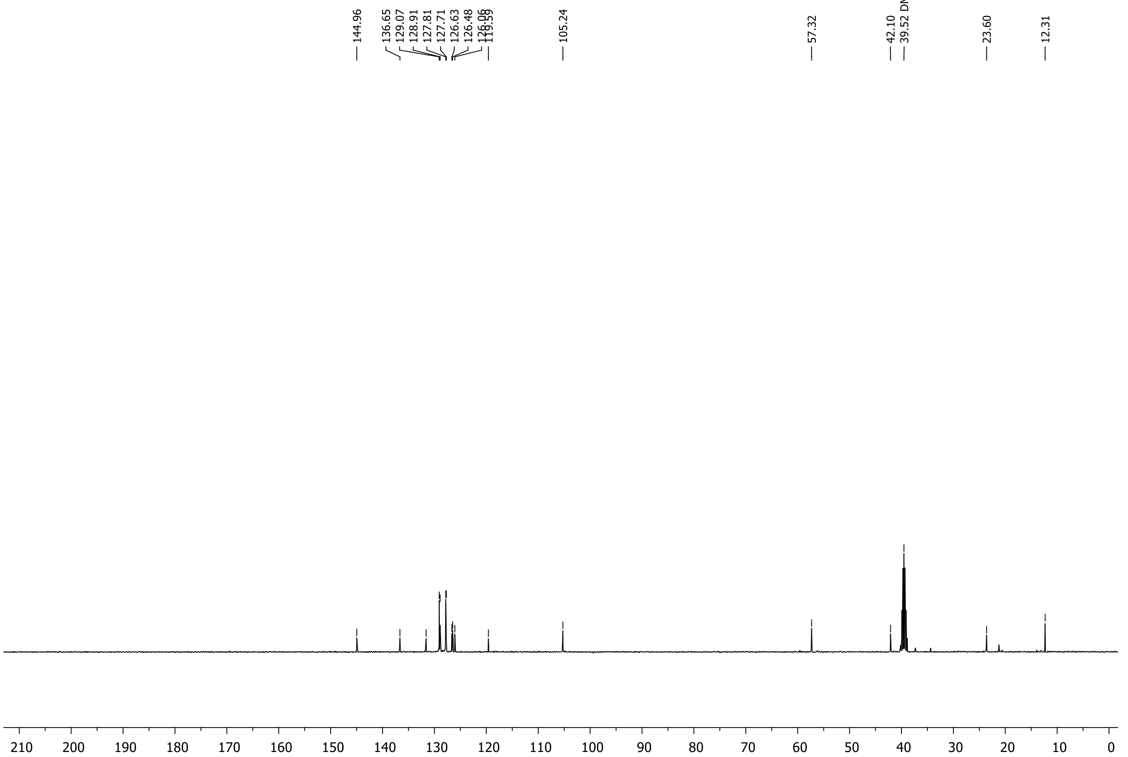

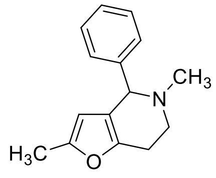

4r

1H, DMSO, 400 MHz

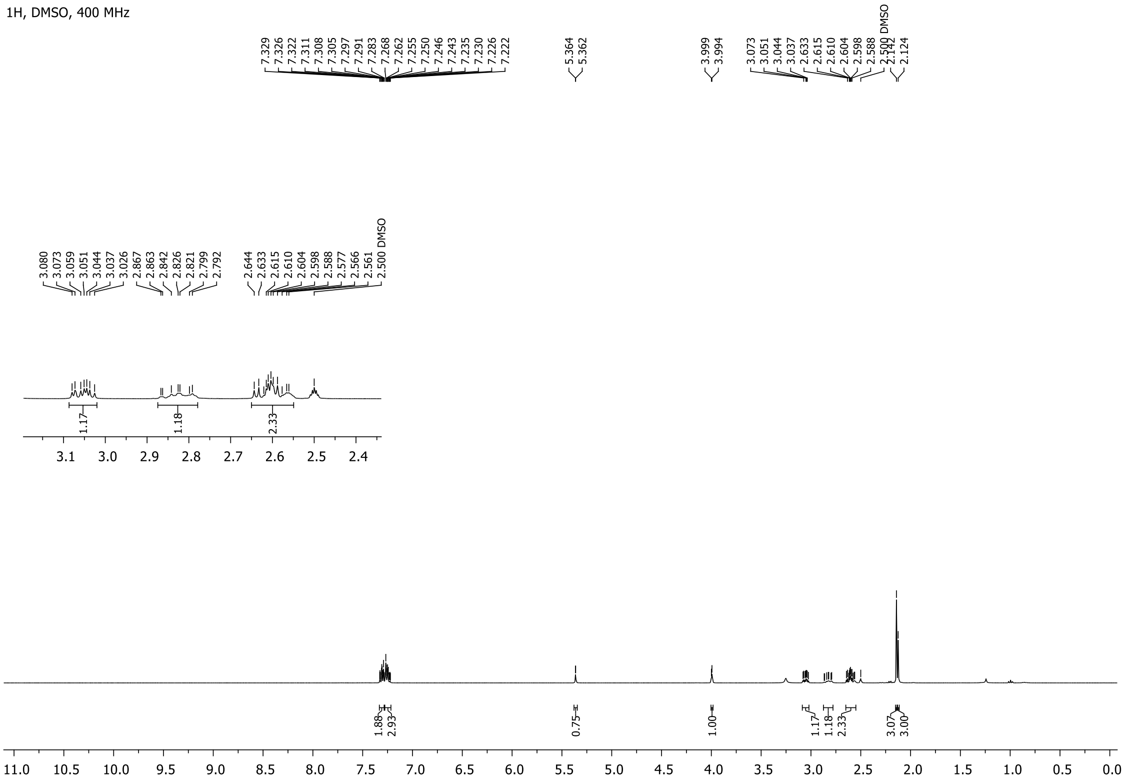

13C, DMSO, 100 MHz

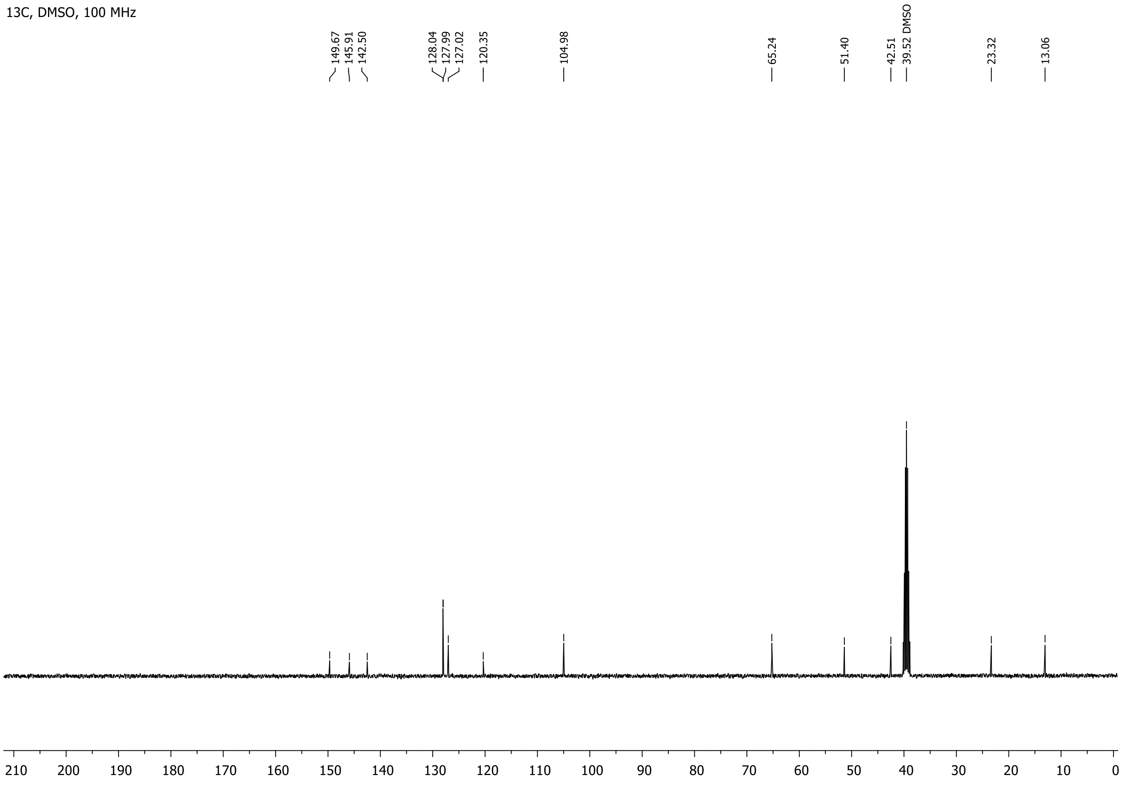

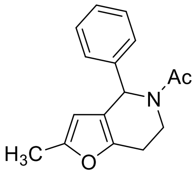

4q

1H, DMSO, 400 MHz

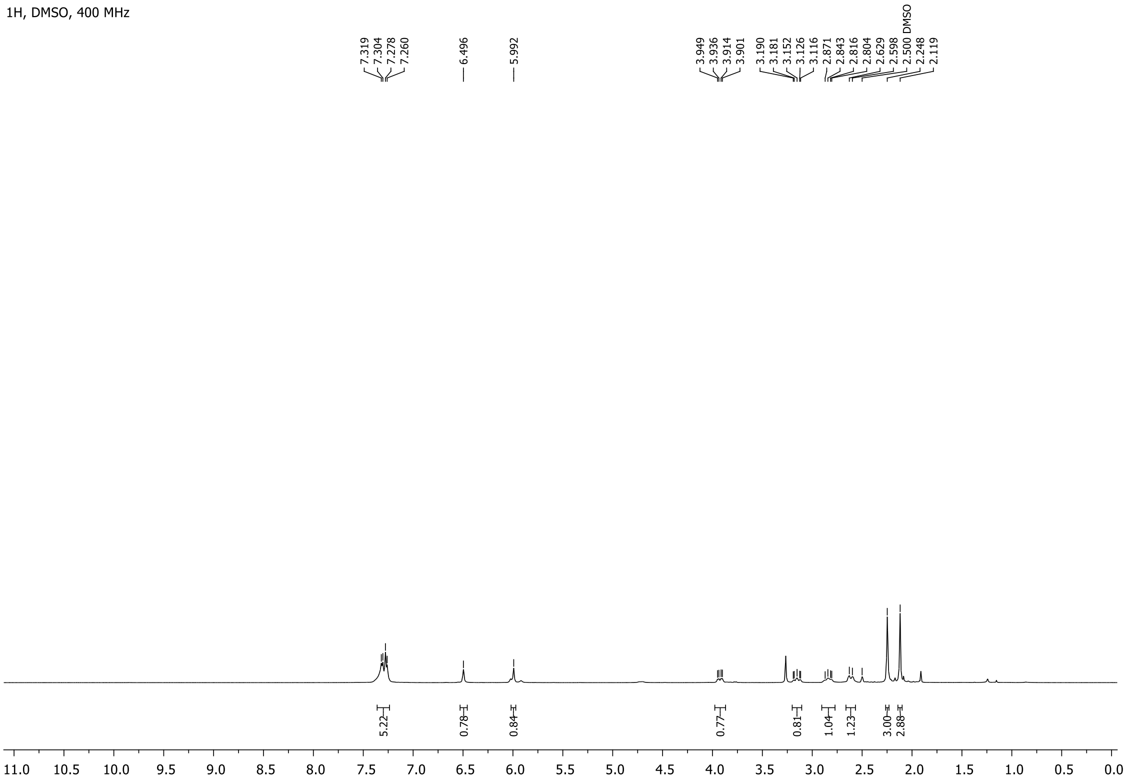

13C, DMSO, 100 MHz

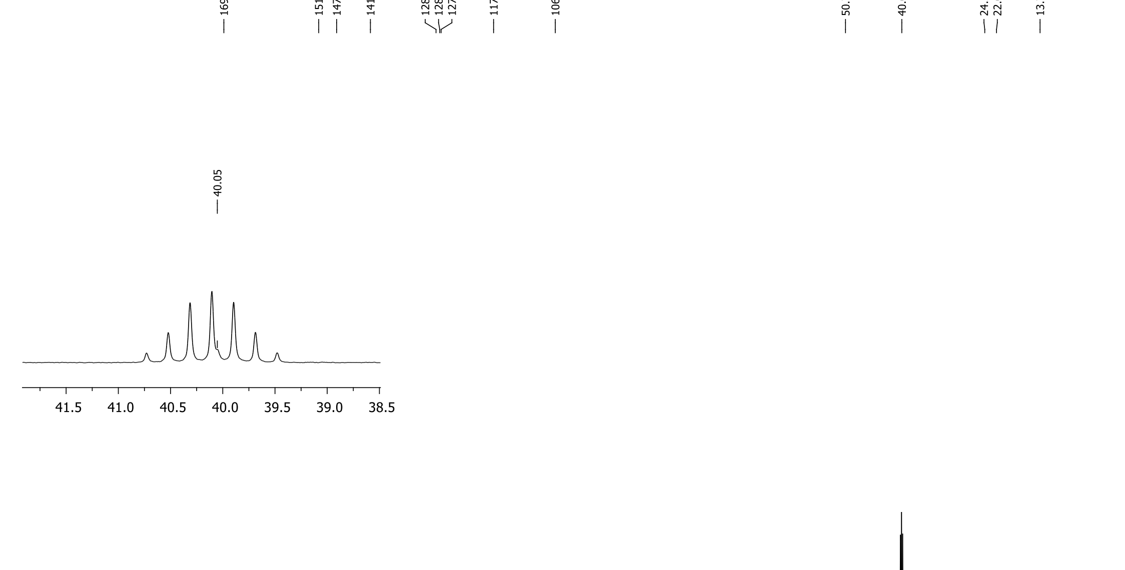

8. X-ray crystallography data

Table S1: Experimental details for 2-methyl-4-phenyl-4,5,6,7-tetrahydrofuro[3,2-*c*]pyridine (**4a**, CCDC 2253942).

Crystal data	
Chemical formula	$C_{14}H_{15}NO$
M_r	213.27
Crystal system, space group	Monoclinic, $P2_1/c$
Temperature (K)	295
a, *b*, *c* (Å)	12.073 (3), 5.7407 (16), 16.957 (4)
β (°)	93.28 (2)
V (Å^3)	1173.3 (5)
Z	4
Radiation type	Mo *K*α
μ (mm^{-1})	0.08
Crystal size (mm)	0.6 × 0.4 × 0.12
Data collection	
Diffractometer	New Xcalibur, Ruby
Absorption correction	Multi-scan<br>*CrysAlis PRO*, Agilent Technologies, Version 1.171.37.33 (release 27-03-2014 CrysAlis171 .NET) (compiled Mar 27 2014,17:12:48) Empirical absorption correction using spherical harmonics, implemented in SCALE3 ABSPACK scaling algorithm.
T_{min}, T_{max}	0.517, 1.000
No. of measured, independent and observed [$I > 2\sigma(I)$] reflections	5659, 2777, 1712
R_{int}	0.043
$(\sin \theta/\lambda)_{max}$ (Å^{-1})	0.693
Refinement	
$R[F^2 > 2\sigma(F^2)]$, $wR(F^2)$, S	0.059, 0.179, 1.03
No. of reflections	2777
No. of parameters	150
H-atom treatment	H atoms treated by a mixture of independent and constrained refinement
$\Delta\rho_{max}$, $\Delta\rho_{min}$ (e Å^{-3})	0.18, −0.28

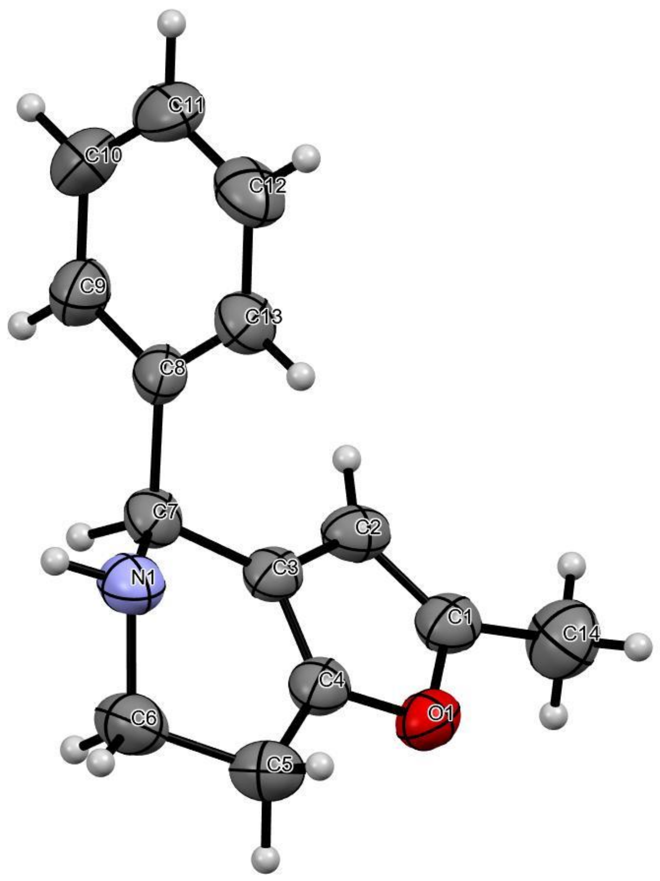

Figure S1: Structure of 2-methyl-4-phenyl-4,5,6,7-tetrahydrofuro[3,2-*c*]pyridine (**4a**, CCDC 2253942) according to the X-ray diffraction data; non-hydrogen atoms are shown as thermal vibration ellipsoids with a probability of 50%.

9. References

1. Demircan, A.; Parsons, P. J. *Heterocycl. Commun.* **2002**, *8*, 531-536. doi: 10.1515/HC.2002.8.6.531
2. Schneider, C. 4-Phenyl-tetrahydro-furano-pyridines and anti-depressant pharmaceutical composition containing same. U.S. Patent 4,636,510, Jan. 13, 1987.
3. Zelina, E. Y.; Nevolina, T. A.; Skvortsov, D. A.; Trushkov, I. V.; Uchuskin, M. G. *J. Org. Chem.* **2019**, *84*, 13707-13720. doi: 10.1021/acs.joc.9b01925
